# KEAP1 Mutations Drive Tumorigenesis by Suppressing SOX9 Ubiquitination and Degradation

**DOI:** 10.1002/advs.202001018

**Published:** 2020-09-27

**Authors:** Na Shao, Hong Huang, Muhammad Idris, Xu Peng, Feng Xu, Shiwu Dong, Chungang Liu

**Affiliations:** ^1^ Department of Biomedical Materials Science School of Biomedical Engineering Army Medical University Chongqing 400038 P.R. China; ^2^ Center of Biological Therapy Southwest Hospital Army Medical University Chongqing 400038 P.R. China; ^3^ Institute of Molecular and Cell Biology Agency for Science Technology and Research (A:STAR) Singapore Singapore

**Keywords:** CKI*γ*, Cullin 3, DNA damage, KEPA1, mutations, SOX9

## Abstract

The transcription factor SOX9 is frequently amplified in diverse advanced‐stage human tumors. Its stability has been shown to be tightly controlled by ubiquitination‐dependent proteasome degradation. However, the exact underlying molecular mechanisms remain unclear. This work reports that SOX9 protein abundance is regulated by the Cullin 3‐based ubiquitin ligase KEAP1 via proteasome‐mediated degradation. Loss‐of‐function mutations in KEAP1 compromise polyubiquitination‐mediated SOX9 degradation, leading to increased protein levels, which facilitate tumorigenesis. Moreover, the loss of critical ubiquitination residues in SOX9, by either a SOX9 (ΔK2) truncation or K249R mutation, leads to elevated protein stability. Furthermore, it is shown that the KEAP1/SOX9 interaction is modulated by CKI*γ*‐mediated phosphorylation. Importantly, it is demonstrated that DNA damage drugs, topoisomerase inhibitors, can trigger CKI activation to restore the KEAP1/SOX9 interaction and its consequent degradation. Collectively, herein the findings uncover a novel molecular mechanism through which SOX9 protein stability is negatively regulated by KEAP1 to control tumorigenesis. Thus, these results suggest that mitigating SOX9 resistance to KEAP1‐mediated degradation can represent a novel therapeutic strategy for cancers with KEAP1 mutations.

## Graphical abstract

This work reports that the Cullin 3^KEAP1^ E3 ubiquitin ligase plays a critical tumor‐suppressive role in lung cancer and liver cancer by negatively controlling SOX9 stability. Therefore, either KEAP1 or SOX9 mutation can lead to elevated SOX9 protein levels by evading the KEAP1‐mediated degradation pathway to promote tumorigenesis.

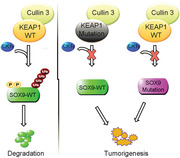

## 1 Introduction

SOX9 is a member of the structurally related sex‐determining region Y (SRY) box containing (SOX) family of transcription factors essential for carrying out diverse functions during development.^[^ ^1^, ^2^, ^3^ ^]^ Heterozygous deletion of SOX9 in mice cause perinatal death, whereas homozygous mice die during embryogenesis.^[^ ^4^, ^5^ ^]^ Further, this protein has been identified as a therapeutic target based on CRISPR‐Cas9 screens of human cancers.^[^ ^6^ ^]^ Both the phosphorylation (by protein kinases A and G at S64 and S181) and SUMOylation of SOX9 protein have been reported to positively affect SOX9 transactivation in chondrocytes and during neural crest development.^[^ ^7^, ^8^ ^]^ Furthermore, SOX9 activity, expression, and localization are regulated by posttranslational modifications including ubiquitylation.^[^ ^9^ ^]^ A pivotal E3 ubiquitin ligase FBW7 recognizes a conserved SOX9 degron phosphorylated by GSK3 and targets it for ubiquitylation and proteasomal degradation.^[^ ^10^, ^11^ ^]^ The tight regulation of SOX9 protein stability is of great importance as even a slight increase in its protein levels can be tremendously consequential, resulting in tumor initiation and progression, with hepatocellular carcinoma (HCC) and lung carcinoma being the most sensitive to such alterations.^[^ ^12^, ^13^, ^14^, ^15^ ^]^ However, little is known about how SOX9 protein stability is governed physiologically by E3 ligase(s) in vivo and how it is aberrantly regulated in HCC and lung carcinoma.

The ubiquitin proteasome system (UPS) represents the major route through which the cells degrade unwanted proteins, with E3 ubiquitin ligases playing a crucial role in conferring specificity to this process.^[^ ^16^ ^]^ The Cullin‐RING E3 ligases represent the largest E3 sub‐family. Further, approximately 600 E3 ubiquitin ligases encoded by the human genome confer substrate specificity to the UPS. In most cases, this is based on the fact that E3s bind their substrates through the recognition of specific short peptide motifs termed “degrons” that control downstream substrate protein stability.^[^ ^17^, ^18^ ^]^ Previous studies have shown that a subset of proteins containing BTB domains comprise substrate‐specific adaptors, which preferentially bind Cullin 3 (CUL3),^[^ ^19^ ^]^ with Kelch‐like ECH‐associated protein 1 (KEAP1) being the first reported mammalian adaptor of the CUL3‐based E3 ligase system.^[^ ^20^ ^]^ KEAP1 is a member of the BTB‐Kelch protein family, and the Kelch domain is responsible for substrate recognition and interaction, whereas the BTB domain binds CUL3, forming the functional E3 ubiquitin ligase complex. Several KEAP1 substrates have been identified, including NRF2, p62, and MGM3, which are associated with proteasome‐dependent degradation.^[^ ^21^, ^22^ ^]^ Furthermore, KEAP1 mutations, deletions, or epigenetic silencing are frequently observed in various cancers.^[^ ^23^, ^24^, ^25^, ^26^ ^]^ Recently, systematic sequencing studies have also revealed that the loss‐of‐function somatic KEAP1 mutations are most frequently found in lung carcinoma (11%) and HCC (8%),^[^ ^23^, ^27^, ^28^ ^]^ indicating a tumor‐suppressive role of this protein in these cancers. Therefore, the identification of additional KEAP1 substrates would benefit clinical diagnosis and therapy.

In the current study, we found that KEAP1 is a novel E3 ligase for SOX9. A significant portion of cancer‐associated mutations in KEAP1 inhibits its ubiquitin ligase activity toward SOX9 and these mutations promote cancer cell growth and tumorigenesis through the stabilization of SOX9. Given the critical oncogenic role of SOX9 and the high frequency of KEAP1 mutations in lung carcinoma and HCC, our study suggests an optimal treatment strategy based on genetic status, which may provide stratified clinical treatments for individual lung carcinoma or HCC patients.

## 2 Results

### 2.1 The CUL3^KEAP1^ E3 Ubiquitin Ligase Negatively Regulates SOX9 Stability

**Figure 1:**
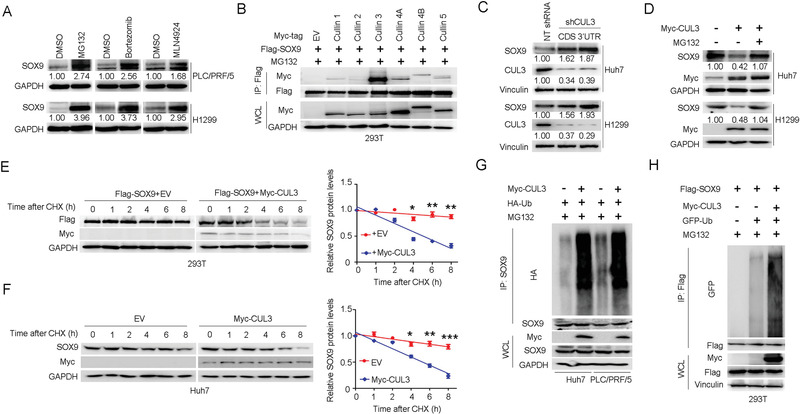
CUL3 targets SOX9 for ubiquitination and degradation. A) Immunoblotting (IB) analysis of SOX9 protein levels in PLC/PRF/5 and H1299 cells after treatment of 20 × 10^−6^ m MG132, 10 × 10^−6^ m bortezomib, or 5 × 10^−6^ m MLN4942 for 6 h. B) IB analysis of WCL and immunoprecipitates (IP) from HEK293T cells transfected with empty vector (EV) or vectors encoding Flag‐tagged SOX9 (Flag‐SOX9) and various Myc‐tagged Cullin constructs (Cullin number is indicated above each column); 30 h after transfection, cells were treated with 20 × 10^−6^ m MG132 for 6 h before harvesting. C) IB analysis of SOX9 protein levels in Huh7 and H1299 cells infected with indicated lentiviral shRNAs targeting CUL3. Cells were selected with 1 µg mL^−1^ puromycin for 72 h to eliminate uninfected cells before harvesting. D) IB analysis of SOX9 protein levels in Huh7 and H1299 cells overexpressing Myc‐CUL3. A total of 36 h after plasmid transfection, cells were treated with 20 × 10^−6^ m MG132 for 6 h before harvesting. E,F) Protein half‐life assay was performed for the assessment of SOX9 stability in HEK293T cells E) or Huh7 cells F) overexpressing Myc‐CUL3. A total of 36 h after plasmid transfection, cells were treated with cycloheximide (CHX, 10 µg mL^−1^) for the indicated time period before they were harvested for IB analyses. Quantification of SOX9 levels relative to GAPDH was shown. Data are presented as mean ± SEM. *n* = 3 independent experiments. ^:^ *p* < 0.05, ^::^ *p* < 0.01, ^:::^ *p* < 0.001, Student's *t* test. G,H) In vivo ubiquitination assay of SOX9 in Huh7 and PLC/PRF/5 cells G) or HEK293T cells H) expressing Myc‐CUL3. A total of 36 h after plasmid transfection, cells were treated with 20 × 10^−6^ m MG132 for 6 h before they were harvested.

Given the prevalence and critical role of SOX9 in cancer progression,^[^ ^6^ ^]^ it is crucial to understand how SOX9 protein stability is regulated and whether the dysregulation of SOX9 protein abundance contributes to cellular resistance to therapy. To this end, we observed that treatment with the proteasome inhibitor MG132 or bortezomib or the Cullin‐RING ubiquitin ligase inhibitor MLN4924 led to marked increases in endogenous SOX9 abundance, indicating the involvement of Cullin‐based ligase(s) in the regulation of SOX9 protein stability (**Figure** **1**A). In support of this notion, we found that SOX9 primarily interacts with CUL3 and to a much lesser extent, the other Cullin family members (Figure 1B). Further, the ectopic expression of CUL1 and CUL3, but not CUL2, CUL4A, CUL4B, or CUL5, decreased the abundance of SOX9 (Figure S1A, Supporting Information). These results indicate that in addition to CUL1/FBW7,^[^ ^10^, ^11^ ^]^ CUL3‐based E3 ligase(s) also play a role in regulating SOX9 stability. Consistent with this notion, depletion of CUL3 elevated the protein abundance of endogenous SOX9 (Figure 1C), and the CUL3‐mediated degradation of SOX9 could be efficiently blocked by MG132 (Figure 1D and Figure S1B, Supporting Information). In contrast, ectopic CUL3 expression decreased the abundance of SOX9 in a dose‐dependent manner (Figure S1C, Supporting Information). More importantly, the half‐life of SOX9 was markedly shortened upon CUL3 overexpression (Figure 1E,F), which was accompanied by with an increase in SOX9 ubiquitination (Figure 1G,H). Collectively, these data suggest that CUL3 plays a critical role in decreasing SOX9 protein abundance, which is associated with HCC and lung carcinoma progression.

**Figure 2:**
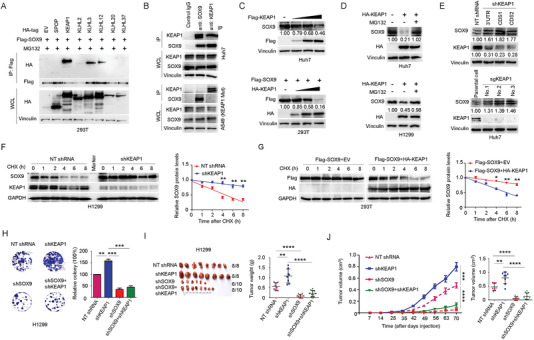
The CUL3^KEAP1^ E3 ubiquitin ligase governs the stability of SOX9 protein. A) Assessment of the binding affinity between SOX9 and CUL3‐based E3 ligase adaptor proteins by immunoblotting (IB) analysis of SOX9 immunoprecipitates (IP) in HEK293T cells expressing HA‐tagged various constructs. A total of 30 h after transfection, cells were treated with 20 × 10^−6^ m MG132 for 6 h before harvesting. B) Co‐IP analysis of SOX9‐KEAP1 interaction in Huh7 and A549 (KEAP1 mutant) cells. Cells were treated with 20 × 10^−6^ m MG132 for 6 h before harvesting. C) IB analysis of SOX9 protein levels in Huh7 and HEK293T cells expressing increasing amount of Flag‐KEAP1. Cells were harvested 48 h after transfection with indicated plasmids. D) IB analysis of SOX9 protein levels in Huh7 and H1299 cells transfected with HA‐KEAP1 plasmid. A total of 36 h after transfection, cells were treated with 20 × 10^−6^ m MG132 for 6 h before harvesting. E) IB analysis of SOX9 protein levels in Huh7 cells upon KEAP1 depletion by shRNA knockdown or CRISPR/Cas9 knockout. Parental Huh7 cells were used as the control. F,G) Protein half‐life assay was performed for the assessment of SOX9 stability in H1299 cells F) or HEK293T cells G) with KEAP1 knockdown F) or overexpression G), respectively. A total of 36 h after plasmid transfection, cells were treated with cycloheximide (CHX, 10 µg mL^−1^) for the indicated time period before they were harvested for IB analyses. Quantification of SOX9 levels relative to GAPDH was shown. Data are presented as mean ± SEM. *n* = 3 independent experiments. ^:^ *p* < 0.05, ^::^ *p* < 0.01, Student's *t* test. H) Colony‐formation assay of H1299 cells with KEAP1 and/or SOX9 shRNA knockdown. Data are presented as mean ± SEM of three independent experiments. ^::^ *p* < 0.01, ^:::^ *p* < 0.001, Student's *t* test. I) Assessment of subcutaneous tumor formation from H1299 cells with KEAP1 and/or SOX9 shRNA knockdown. Tumor mass was measured at the endpoint of the study. Data are presented as mean ± SEM, *n* = 8 or 10 per group. ^::^ *p* < 0.01, ^::::^ *p* < 0.0001, Student's *t* test. J) In vivo subcutaneous tumor growth from H1299 cells with KEAP1 and/or SOX9 shRNA knockdown was monitored over the indicated time period (left panel). Tumor volumes at the endpoint of the study were shown in the right panel. Data are presented as mean ± SEM, *n* = 8 or 10 per group. ^::^ *p* < 0.01, ^::::^ *p* < 0.0001, Student's *t* test.

CUL3‐based E3 ubiquitin ligases recognize their downstream substrates through substrate‐recruiting adaptor proteins.^[^ ^29^ ^]^ We found that KEAP1, and to a lesser extent KLHL3 and KLHL12, but not other examined adaptor proteins, interact with SOX9 (**Figure** **2**A). Moreover, a functionally null KEAP1 mutant showed no physical binding to SOX9 (Figure 2B). Consistent with the observation of SOX9‐KEAP1 interaction in the Co‐IP assay in Huh7 cell lysate, we also found that KEAP1 colocalized with SOX9 in the cytoplasm of Huh7 cells through the immunofluorescence (IF) staining assay (Figure S2A, Supporting Information). Notably, KEAP1, but not SPOP, KLHL3, or KLHL12, promoted SOX9 protein degradation in a dose‐dependent manner (Figure 2C and Figure S2B,C, Supporting Information). Moreover, the KEAP1‐mediated degradation of SOX9 could be efficiently blocked by MG132 (Figure 2D and Figure S2D, Supporting Information), indicating that KEAP1 regulates SOX9 protein abundance through the ubiquitin‐proteasome pathway. In accordance with these findings, the depletion of endogenous KEAP1 by short hairpin RNA (shRNA)‐mediated knockdown or CRISPR‐mediated knockout in multiple liver and lung cancer cell lines led to a marked increase in the abundance of SOX9 protein (Figure 2E and Figure S2E, Supporting Information), but not its mRNA levels (Figure S2F, Supporting Information). Notably, the half‐life of SOX9 was markedly extended or shortened in KEAP1‐depleted or overexpressed cells (Figure 2F,G and Figure S2G, Supporting Information), respectively.

NRF2 transcription factor is a major target of KEAP1‐mediated degradation, and NRF2 promotes SOX9 mRNA expression in other systems, such as urethane‐induced murine lung carcinogenesis model, podocytes, and bovine articular chondrocytes.^[^ ^30^, ^31^, ^32^ ^]^ We asked whether KEAP1‐mediated downregulation of SOX9 protein levels was a consequence of NRF2 degradation through transcriptional regulation. We found that silencing NRF2 through shRNA had no obvious effect on SOX9 mRNA levels in human lung cancer cell line‐H1299, as determined by qRT‐PCR (Figure S2F, Supporting Information). Furthermore, the lung carcinoma datasets from The Cancer Genome Atlas (TCGA) showed that NRF2 or KEAP1 mRNA levels do not correlate with SOX9 mRNA levels (Figure S2H, Supporting Information). These results suggest that SOX9 protein level is regulated through a posttranslational mechanism, potentially by the CUL3^KEAP1^ E3 ubiquitin ligase.

Next, we sought to understand the biological role of KEAP1 in governing SOX9 stability. SOX9 has been previously shown to play a critical role in cell proliferation and tumorigenesis.^[^ ^10^, ^12^, ^14^, ^15^ ^]^ In agreement with previous studies, we observed that, in comparison with the control (NT shRNA), the depletion of SOX9 (shSOX9) significantly inhibited colony formation in H1299 and Huh7 cells (Figure 2H and Figure S2I,J, Supporting Information) and tumorigenesis (Figure 2I,J). In contrast, the depletion of endogenous KEAP1 led to a marked elevation in SOX9 protein abundance (Figure S2I, Supporting Information) and a SOX9‐dependent increase in colony formation (Figure 2H and Figure S2J, Supporting Information) and tumorigenesis (Figure 2I,J) relative to those in controls. Altogether, these data suggest that CUL3^KEAP1^ suppresses cancer progression largely through promoting Sox9 poly‐ubiquitination and degradation in the HCC and lung carcinoma setting.

### 2.2 KEAP1 Negatively Regulates SOX9 Protein Stability in a Poly‐Ubiquitination Dependent Manner

**Figure 3:**
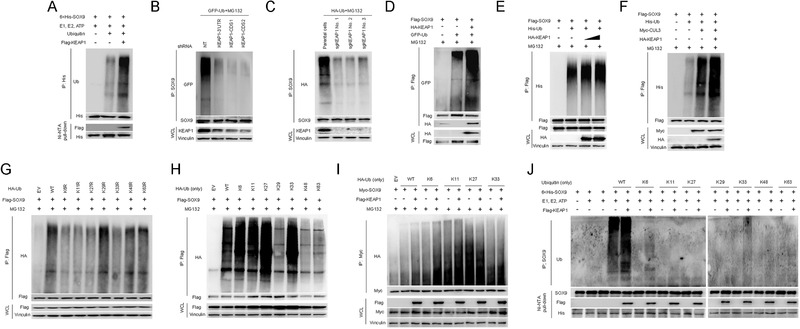
KEAP1 poly‐ubiquitinates SOX9. A) In vitro SOX9 ubiquitination assay by KEAP1. Purified 6 × His‐SOX9 was incubated with or without Flag‐KEAP1 in the presence of required components of ubiquitination system including HA‐RBX1, Myc‐Cullin 3, E1, UbcH5C, UBE1, and ubiquitin for 2 h at 37 °C. After in vitro ubiquitination reaction, SOX9 was immunoprecipitated anti‐SOX9 antibody and immunoblotted with anti‐ubiquitin antibody. Ub, ubiquitin. B,C) Effects of KEAP1 knockdown by shRNAs in H1299 cells B) or knockout by CRISPR‐Cas9 in Huh7 cells C) on SOX9 ubiquitination were evaluated by immunoblotting (IB) analysis. KEAP1 shRNA transfected H1299 cells and KEAP1‐KO Huh7 cells were treated with 20 × 10^−6^ m MG132 for 6 h before harvesting. SOX9 was immunoprecipitated with anti‐SOX9 antibody and immunoblotted with anti‐GFP or anti‐HA antibody which specifically recognizes GFP‐ or HA‐tagged ubiquitin. HA‐Ub or GFP‐Ub, HA‐ or GFP‐tagged ubiquitin. D−F) In vivo ubiquitination analysis of SOX9 in HEK293T cells overexpressing HA‐KEAP1 D,E) and Myc‐CUL3 F). A total of 36 h after plasmid transfection, cells were treated with 20 × 10^−6^ m MG132 for 6 h before they were harvested. Flag‐SOX9 was immunoprecipitated with anti‐Flag antibody and immunoblotted with anti‐GFP or anti‐His antibody which specifically recognizes GFP‐ or His‐tagged ubiquitin. G) SOX9 poly‐ubiquitination linkage was examined by transfecting HA‐tagged wild‐type (WT) or indicated ubiquitin mutants containing point mutations of lysine 6, 11, 27, 29, 33, 48, or 63 to arginine together with Flag‐SOX9 into HEK293T cells, followed by IB analysis of HA‐Ub in anti‐Flag IP products. Cells were treated with 20 × 10^−6^ m MG‐132 for 6 h before harvesting. H) SOX9 poly‐ubiquitination linkage was examined by transfecting HA‐tagged WT or indicated ubiquitin mutants containing Lys 6/11/27/29/33/48/63‐only mutations (the other six of seven lysine residues were mutated to arginine) together with Flag‐SOX9 into HEK293T cells, followed by IB analysis of HA‐Ub in anti‐Flag IP products. Cells were treated with 20 × 10^−6^ m MG‐132 for 6 h before harvesting. I) Effects of KEAP1 overexpression on SOX9 poly‐ubiquitination in HEK293T cells transfected with the indicated ubiquitin Lys 6/11/27/33‐only mutant plasmids. A total of 36 h after transfection, cells were treated with 20 × 10^−6^ m MG132 for 6 h before they were harvested for Myc‐tag IP and HA‐Ub IB analyses. J) In vitro SOX9 ubiquitination linkage assay. Purified 6 × His‐SOX9 was incubated with or without Flag‐KEAP1 in the presence of essential components of ubiquitination system including HA‐RBX1, Myc‐Cullin 3, E1, UbcH5C, UBE1, and WT or mutant ubiquitin for 2 h at 37 °C. After reaction, SOX9 was immunoprecipitated with anti‐SOX9 antibody and immunoblotted with anti‐ubiquitin antibody.

As KEAP1 is a ubiquitylase and it regulates SOX9 stability, we first asked whether SOX9 is a direct target of its enzymatic activity. Indeed, in vitro ubiquitylation assays showed that KEAP1 directly adds the ubiquitin chain to SOX9 (**Figure** **3**A). Further, the downregulation or knockout of KEAP1 decreased SOX9 ubiquitylation in H1299 or Huh7 cells (Figure 3B,C). Conversely, ectopic expression of KEAP1 promoted SOX9 ubiquitylation in a dose‐dependent manner (Figure 3D,E). The overexpression of CUL3 further promoted KEAP1 in triggering SOX9 poly‐ubiquitination (Figure 3F).

Protein ubiquitination controls protein stability, trafficking, and protein−protein interactions via seven possible linkages of poly‐ubiquitin chains.^[^ ^33^ ^]^ We therefore sought to determine the type of ubiquitin chain that was added to SOX9 by KEAP1.We mutated each of the lysine residues (Figure 3G) or six of the seven lysine residues to arginine in ubiquitin (Figure 3H) to examine the levels of SOX9 poly‐ubiquitination in KEAP1‐overexpressing cells. Results showed that KEAP1 efficiently added Lys 6, Lys 11, Lys 27, and Lys 33 types of ubiquitin chains onto SOX9 (Figure 3G−I). To extend our findings, we performed a thorough in vitro SOX9 ubiquitylation assay with KEAP1 based on a series of ubiquitin mutants. Surprisingly, results showed that KEAP1 efficiently added Lys 6 and Lys 33, but not Lys 11 and Lys 27 types of ubiquitin chains on SOX9 in vitro (Figure 3J), suggesting that posttranslational modifications and/or additional proteins are required for KEAP1 to act on SOX9 in vivo. Taken together, our studies showed that KEAP1 is a specific ubiquitylase that polyubiquitylates and destabilizes SOX9.

### 2.3 Cancer‐Associated KEAP1 Mutants Promote Tumorigenesis by Elevating SOX9 Protein Levels

**Figure 4:**
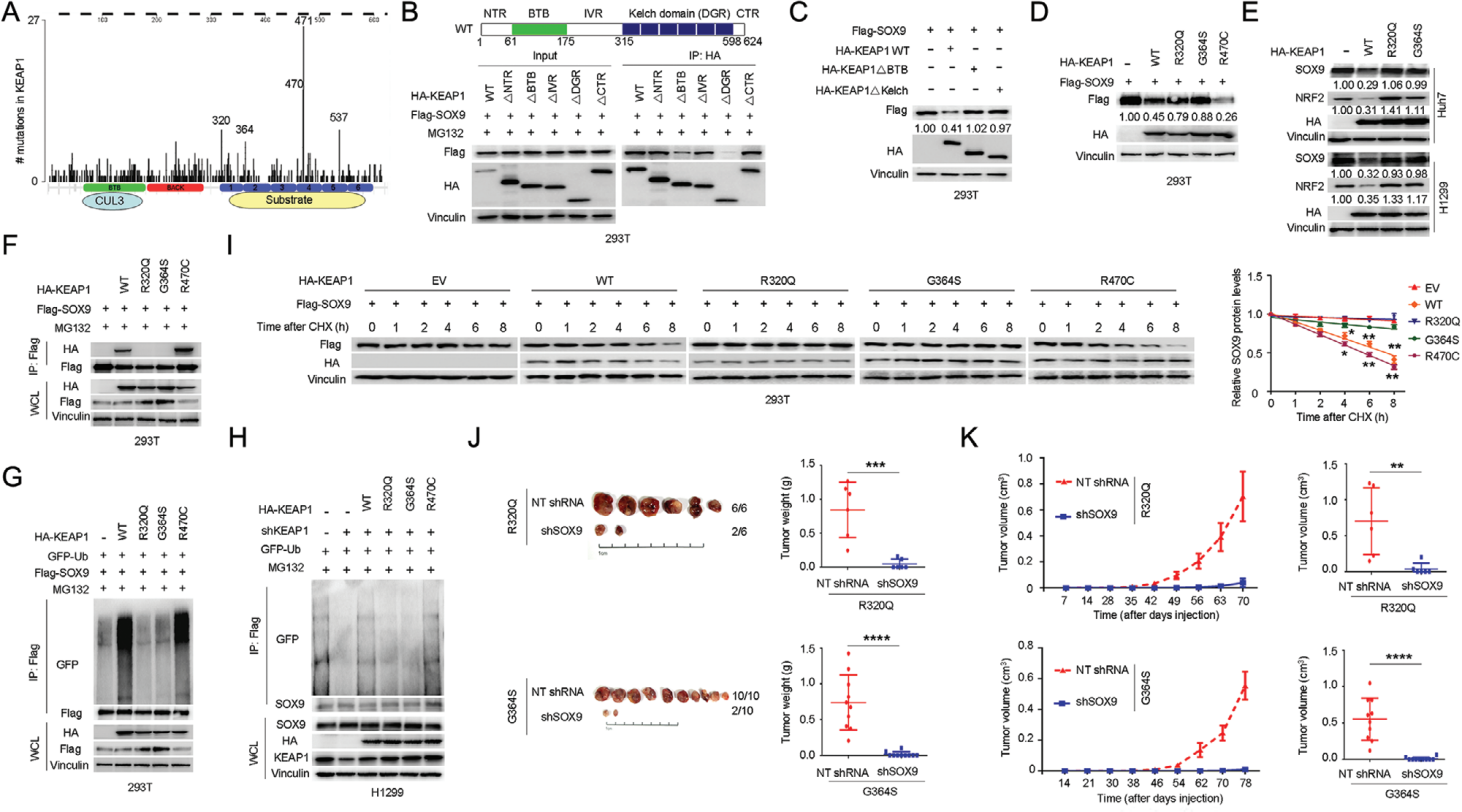
Cancer‐associated KEAP1 mutants promote tumorigenesis by elevating SOX9 protein levels. A) Schematic of KEAP1 functional domains and cancer‐associated mutations identified previously. B) Schematic diagram showing the functional domains of KEAP1 (top panel). HA‐tagged full‐length or truncation mutants of KEAP1 were coexpressed with Flag‐SOX9 in 293T cells. A total of 36 h after plasmid transfection, cells were treated with 20 × 10^−6^ m MG132 for 6 h before harvesting. Full‐length and truncated HA‐KEAP1 were immunoprecipitated with HA antibody, and the binding of Flag‐SOX9 was examined by western blotting using Flag antibody. The serial truncation mutants of HA‐KEAP1 are NTR domain deletion (ΔNTR), BTB domain deletion (ΔBTB), IVR domain deletion (ΔIVR), DGR domain deletion (ΔDGR), and CTR domain deletion (ΔCTR). C) Immunoblotting (IB) analysis of SOX9 protein levels in HEK293T cells expressing HA‐tagged wild‐type (WT) KEAP1 or mutant KEAP1 with BTB domain deletion (ΔBTB) or Kelch domain deletion (ΔKelch). Cells were harvested 48 h after transfection with indicated plasmids. D) IB analysis of SOX9 protein levels in HEK293T cells transfected with the indicated cancer‐associated KEAP1 mutants. Cells were harvested 48 h after transfection with indicated plasmids. E) IB analysis of SOX9 and NRF2 protein levels in Huh7 and H1299 cells stably expressing HA‐tagged WT‐KEAP1 or the indicated cancer‐associated KEAP1 mutants. F) Co‐IP analysis of SOX9‐KEAP1 interaction in HEK293T cells expressing HA‐tagged WT KEAP1 or the indicated cancer‐associated KEAP1 mutants. A total of 36 h after transfection, cells were treated with 20 × 10^−6^ m MG132 for 6 h before harvesting. G,H) In vivo ubiquitination analysis of SOX9 in HEK293T cells G) and H1299 cells H) expressing the indicated cancer‐associated KEAP1 mutants. A total of 36 h after plasmid transfection, cells were treated with 20 × 10^−6^ m MG132 for 6 h before they were harvested. I) Protein half‐life assay was performed for the assessment of SOX9 stability in HEK293T cells expressing the indicated cancer‐associated KEAP1 mutants. A total of 36 h after plasmid transfection, cells were treated with cycloheximide (CHX, 10 µg mL^−1^) for the indicated time period before they were harvested for IB analyses. Quantification of SOX9 levels relative to GAPDH was shown. Data are presented as mean ± SEM. *n* = 3 independent experiments. ^:^ *p* < 0.05, ^::^ *p* < 0.01, Student's *t* test. J) Assessment of subcutaneous tumor formation from H1299 cells stably expressing the cancer‐associated R320Q or G364S mutant KEAP1 with or without SOX9 shRNA knockdown. Tumor weight was measured at the endpoint of the study. Data are presented as mean ± SEM, *n* = 6 or 10 per group. ^:::^ *p* < 0.001, ^::::^ *p* < 0.0001, Student's *t* test. K) In vivo subcutaneous tumor growth was monitored over the indicated time period (left panel) from H1299 cells stably expressing the R320Q or G364S mutant KEAP1 with or without SOX9 shRNA knockdown. Tumor volumes at the endpoint of the study were shown in the right panel. Data are presented as mean ± SEM, *n* = 6 or 10 per group. ^::^ *p* < 0.01, ^::::^ *p* < 0.0001, Student's *t* test.

More than 50 different mutations have been mapped to the human KEAP1 gene in various cancers, for which mutations are largely clustered within the BTB and Kelch domain (https://cancer.sanger.ac.uk/cosmic and http://www.cbioportal.org/; **Figure** **4**A and Figure S3A, Supporting Information). To identify the KEAP1 mutations affecting SOX9 stability, we first asked which domains in KEAP1 are required for SOX9 binding and subsequent degradation. In this regard, we generated a series of KEAP1 domain‐deletion mutants and coexpressed them with SOX9 in HEK293T cells. Co‐IP results showed that deletion of the BTB or Kelch domain, but not the NTR, IVR, or CTR domain, prevented KEAP1 binding to SOX9 (Figure 4B). Moreover, loss of either the BTB or Kelch domain inhibits KEAP1‐mediated SOX9 poly‐ubiquitination (Figure S3B, Supporting Information) and degradation (Figure 4C and Figure S3C, Supporting Information). Given that the Kelch domain deletion showed the strongest effect on SOX9 binding, we examined three cancer‐associated KEAP1 mutations within this domain for their effects on SOX9 stability. Notably, two of the KEAP1 mutants R320Q and G364S failed to promote SOX9 and NRF2 degradation (Figure 4D,E and Figure S3D, Supporting Information) due to deficiencies in SOX9 binding (Figure 4F and Figure S3E, Supporting Information) and poly‐ubiquitination (Figure 4G,H). Moreover, the ectopic expression of wide‐type KEAP1 (KEAP1‐WT), but not the R320Q and G364S mutants, substantially shortened the half‐life of SOX9 (Figure 4I) relative to control levels. Interestingly, the KEAP1 R470C mutant exhibited enhanced SOX9 binding in the Co‐IP experiment (Figure 4F); this “superbinder” mutant was shown to behave in a similar way as the WT KEAP1 in promoting SOX9 poly‐ubiquitination (Figure 4G,H and Figure S3F, Supporting Information) and degradation (Figure 4D,I and Figure S3D, Supporting Information).

In line with these findings, we found that the depletion of SOX9 by shRNA‐mediated knockdown in cells expressing KEAP1 R320Q and G364S mutants significantly retarded colony formation (Figure S3G,H, Supporting Information), and tumor growth in xenograft mouse models (Figure 4J,K). Overall, these results suggest a pathogenic role for SOX9 in promoting tumorigenesis downstream of cancer‐specific KEAP1 mutations.

**Figure 5:**
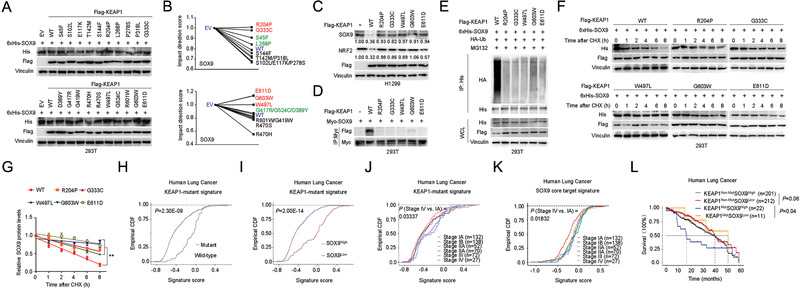
A subset of lung carcinoma associated mutations in KEAP1 reduce its activity in SOX9 binding, poly‐ubiquitination and degradation. A,B) Immunoblotting (IB) analysis of SOX9 protein levels in HEK293T cells transfected with the indicated lung carcinoma‐associated KEAP1 mutants. Cells were harvested 48 h after transfection with the indicated plasmids A). Quantification of SOX9 levels relative to Vinculin was shown in B). C) IB analysis of SOX9 and NRF2 protein levels in H1299 cells transfected with the indicated lung carcinoma‐associated KEAP1 mutants. D) Co‐IP analysis of SOX9‐KEAP1 interaction in HEK293T cells expressing Flag‐tagged wild‐type (WT) KEAP1 or the indicated lung carcinoma‐associated KEAP1 mutants. E) In vivo ubiquitination analysis of SOX9 in HEK293T cells expressing Flag‐tagged WT‐KEAP1 or the indicated lung carcinoma‐associated KEAP1 mutants. A total of 36 h after plasmid transfection, cells were treated with 20 × 10^−6^ m MG132 for 6 h before harvesting. SOX9 was immunoprecipitated with antibody against His tag and probed for ubiquitination status with HA antibody. F,G) Protein half‐life analysis of SOX9 in HEK293T cells expressing the indicated lung carcinoma associated KEAP1 mutants R204P, G333C, W497L, G603W, or E611D F). A total of 36 h after plasmid transfection, cells were treated with cycloheximide (CHX, 10 µg mL^−1^) for the indicated time period before they were harvested for IB analyses. Quantification of SOX9 levels relative to Vinculin was shown G). Data are presented as mean ± SEM. *n* = 3 independent experiments. ^::^ *p* < 0.01, two‐way ANOVA test. H) Empirical cumulative distribution function (ECDF) plots showing expression correction of individual KEAP1‐mutant signature within the The Cancer Genome Atlas (TCGA) lung cancer cohort. I) CDF plots showing expression correlation of individual tumors with the KEAP1‐mutant signature across SOX9 expression levels within the TCGA lung cancer cohort. J,K) CDF plots showing expression correlation of individual tumors with the KEAP1‐mutant signature J) or SOX9 core target signature K) across various clinical stages within the TCGA lung cancer cohort. Each curve represents a unique clinical stage. L) Kaplan−Meier survival curves of lung cancer patients based on KEAP1 mutational status and the relative strength of SOX9 expression. Long‐rank tests (two‐sided) were used for the statistical analysis.

To establish a mechanistic link between SOX9 and the KEAP1 mutations specifically associated with lung carcinoma, we investigated a set of KEAP1 missense mutations identified in this cancer type.^[^ ^34^ ^]^ Toward this end, we constructed a series of plasmids harboring KEAP1 mutants with point‐mutations, of which, 12 mutations are located in the Kelch domain. Intriguingly, five KEAP1 mutants, specifically R204P, G333S, W497L, G603W, and E611D, lost their ability to destabilize the ectopically expressed SOX9 protein in 293T cells (**Figure** **5**A,B). Subsequently, we validated this finding further in the H1299 lung cancer cells by showing the same set of five KEAP1 mutants failed to destabilize endogenous SOX9 (Figure 5C). To investigate the underlying mechanism, we evaluated the interactions between these KEAP1 mutants and SOX9 by Co‐IP assay. In contrast to WT KEAP1, the interaction between KEAP1 mutants (R204P, G333S, W497L, G603W, and E611D) and SOX9 was hardly detectable (Figure 5D), suggesting that these mutations impaired KEAP1 binding to SOX9. Consistently, KEAP1‐R204P, G333S, W497L, G603W, or E611D mutants had no significantly reduced SOX9 poly‐ubiquitination (Figure 5E) and shortened its half‐life (Figure 5F,G). Furthermore, KEAP1 mutations showed significant positive correlation with SOX9 expression in the TCGA lung cancer cohort (Figure 5H,I). In addition, by analyzing the TCGA human lung cancer dataset, we found that the advanced‐stage tumors (clinical stage IV disease) were significantly enriched for the human KEAP1‐mutant transcriptional signature (Figure 5J), and core SOX9 target genes were significantly upregulated in tumors from advanced stage (Figure 5K). While the mutational status of KEAP1 is one of the most critical biomarkers for molecular classification and general survival time of the patients,^[^ ^23^ ^]^ high SOX9 expression appeared to confer poor outcome in KEAP1‐mutated lung cancer patients (Figure 5L). Taken together, these data suggest that a subset of the KEAP1 mutations leads to elevate SOX9 protein level, which is associated with lung cancer progression.

### 2.4 KEAP1 Ubiquitinates SOX9 in a Degron‐Dependent Manner on Lysine 249 to Suppress Oncogenicity

**Figure 6:**
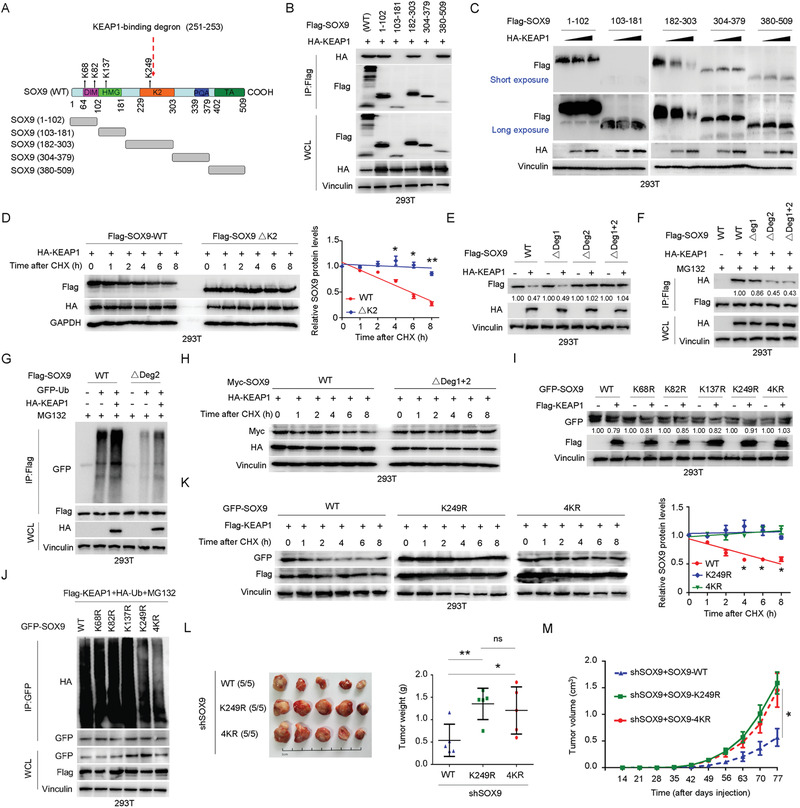
KEAP1 promotes SOX9 lysine‐249 ubiquitination and subsequent degradation in a degron‐dependent manner to suppress its oncogenicity. A) Schematic of SOX9 functional domains, ubiquitination sites, and the truncated SOX9 constructs used in this study. B) Co‐IP analysis of SOX9‐KEAP1 interaction in HEK293T cells expressing Flag‐tagged wild‐type (WT) SOX9 or the indicated truncated SOX9 mutants. C) Immunoblotting (IB) analysis of the protein levels of the indicated truncated SOX9 mutants in HEK293T cells expressing increasing amounts of HA‐KEAP1. D) Protein half‐life analysis of WT‐SOX9 and SOX9 K2 domain deletion mutant (ΔK2) in HEK293T cells expressing KEAP1. A total of 36 h after plasmid transfection, cells were treated with cycloheximide (CHX, 10 µg mL^−1^) for the indicated time period before they were harvested for IB analyses. Quantification of SOX9 levels relative to GAPDH was shown. Data are presented as mean ± SEM. *n* = 3 independent experiments. ^:^ *p* < 0.05, ^::^ *p* < 0.01, Student's *t* test. E) IB analysis of the protein levels of WT‐SOX9 and the indicated SOX9 degron deletion mutants in HEK293T cells with or without ectopic HA‐KEAP1 expression. F) Co‐IP analysis of SOX9‐KEAP1 interaction in HEK293T cells expressing Flag‐tagged WT‐SOX9 or the indicated SOX9 degron deletion mutants. G) In vivo ubiquitination assay of SOX9 in HEK293T cells expressing Flag‐tagged WT‐SOX9 or the SOX9 degron2 deletion mutant (ΔDeg2) in the presence or absence of ectopic KEAP1 expression. A total of 36 h after plasmid transfection, cells were treated with 20 × 10^−6^ m MG132 for 6 h before harvesting. H) Protein half‐life analysis of WT‐SOX9 and SOX9 degron 1 + 2 deletion mutant (ΔDeg1+2) in HEK293T cells expressing KEAP1. A total of 36 h after plasmid transfection, cells were treated with 10 µg mL^−1^ CHX for the indicated time period before they were harvested for IB analyses. I) IB analysis of the protein levels of WT‐SOX9 and the indicated SOX9 K to R mutants in HEK293T cells with or without ectopic Flag‐KEAP1 expression. J) In vivo ubiquitination assay of SOX9 in HEK293T cells expressing GFP‐tagged WT‐SOX9 or the indicated SOX9 K to R mutants in the presence of ectopic KEAP1 expression. A total of 36 h after plasmid transfection, cells were treated with 20 × 10^−6^ m MG132 for 6 h before harvesting. K) Protein half‐life analysis of WT‐SOX9 and the indicated SOX9 K to R mutants in HEK293T cells expressing KEAP1. A total of 36 h after plasmid transfection, cells were treated with 10 µg mL^−1^ CHX for the indicated time period before they were harvested for IB analyses. Quantification of SOX9 levels relative to Vinculin was shown. Data are presented as mean ± SEM. *n* = 3 independent experiments. ^:^ *p* < 0.05, Student's *t* test. L) Assessment of subcutaneous tumor formation from H1299 cells stably expressing SOX9‐WT, SOX9 K249R, or SOX9 4KR mutant with endogenous SOX9 knocked down by shRNA. Tumor weight was measured at the endpoint of the study. Data are presented as mean ± SEM, *n* = 5 per group. ^:^ *p* < 0.05, ^::^ *p* < 0.001, Student's *t* test. M) In vivo tumor growth from the cells described above was monitored over the indicated time period. Data are presented as mean ± SEM, *n* = 5 per group. ^:^ *p* < 0.05, Student's *t* test.

To gain further insights into how KEAP1 governs SOX9 stability, we next examined the specific region(s) of SOX9 that interact with KEAP1 (**Figure** **6**A). Interestingly, KEAP1 interacts with SOX9 at several domains (Figure 6B) but only promotes the degradation of the K2 domain, but not the TA domain or other regions of SOX9 (Figure 6C). Moreover, K2 domain deletion mutant was resistant to KEAP1‐mediated SOX9 poly‐ubiquitination and degradation (Figure S4A,B, Supporting Information), which substantially prolonged the half‐life of SOX9 (Figure 6D), suggesting that the K2 domain of SOX9 is both necessary and sufficient for SOX9 ubiquitination.

A previous study reported that the well‐characterized substrates of KEAP1 contain a DLG or ETEG conserved motif.^[^ ^20^, ^35^, ^36^ ^]^ Upon examination of the primary sequence of SOX9, two highly conserved DLK motifs, resembling the DLG “degron” sequence, were found at the N‐terminal and K2 domain of SOX9 (Figure S4C, Supporting Information). Notably, the deletion of degron 2 (ΔDeg2) within the K2 domain, but not degron 1 (ΔDeg1), largely blocked KEAP1‐mediated SOX9 degradation, whereas SOX9 was no longer subjected to KEAP1‐mediated degradation when both degrons 1 and 2 were deleted (ΔDeg1+2) (Figure 6E and Figure S4D, Supporting Information). These data suggest that degron 2 is the major KEAP1‐binding site, whereas degron 1 plays a dispensable role in KEAP1‐mediated SOX9 degradation. Consistently, compared with the WT, the deletion of degron 1 only moderately reduced, whereas the deletion of degron 2 or both degrons dramatically decreased, SOX9 interaction with KEAP1 in cells (Figure 6F). Moreover, degron 2 deletion prevented KEAP1‐mediated SOX9 poly‐ubiquitination (Figure 6G), which substantially prolonged the half‐life of SOX9 (Figure 6H and Figure S4E, Supporting Information). Next, we explored the physiological roles of degron‐mediated SOX9 degradation by KEAP1 in HCC and lung carcinoma. In this analysis, we found that compared to wide‐type cells, cells expressing the SOX9 nondegradable (ΔDeg 2) mutant showed increased colony formation (Figure S4F, Supporting Information), supporting an oncogenic role for SOX9.

Four lysine residues have previously been identified by mass spectrometry as potential ubiquitination sites in SOX9 (Figure 6A), all of which are evolutionarily conserved. We found that the KEAP1‐mediated poly‐ubiquitination and degradation of SOX9 were blocked if all four lysine (K) residues or the single lysine 249 residue were mutated to arginine (R) (Figure 6I,J), which substantially prolonged the half‐life of SOX9 (Figure 6K). However, none of the other three single mutations (K68R, K82R, and K137R) blocked SOX9 ubiquitination and degradation (Figure 6I,J). Therefore, we conclude that the CUL3^KEAP1^ E3 ligase catalyzes ubiquitination on K249 site within the K2 domain of SOX9 in a degron‐dependent manner. Notably, compared to H1299‐shSOX9 cells expressing WT SOX9, cells expressing the ubiquitination‐deficient SOX9 mutants (4KR or K249R) showed increased tumor growth in xenograft model (Figure 6L,M and Figure S4G, Supporting Information). These data suggest that the loss of SOX9‐ubiquitination is critical for tumorigenicity.

### 2.5 Casein Kinase I Phosphorylates SOX9 to Trigger KEAP1‐SOX9 Interaction and Promote SOX9 Degradation

**Figure 7:**
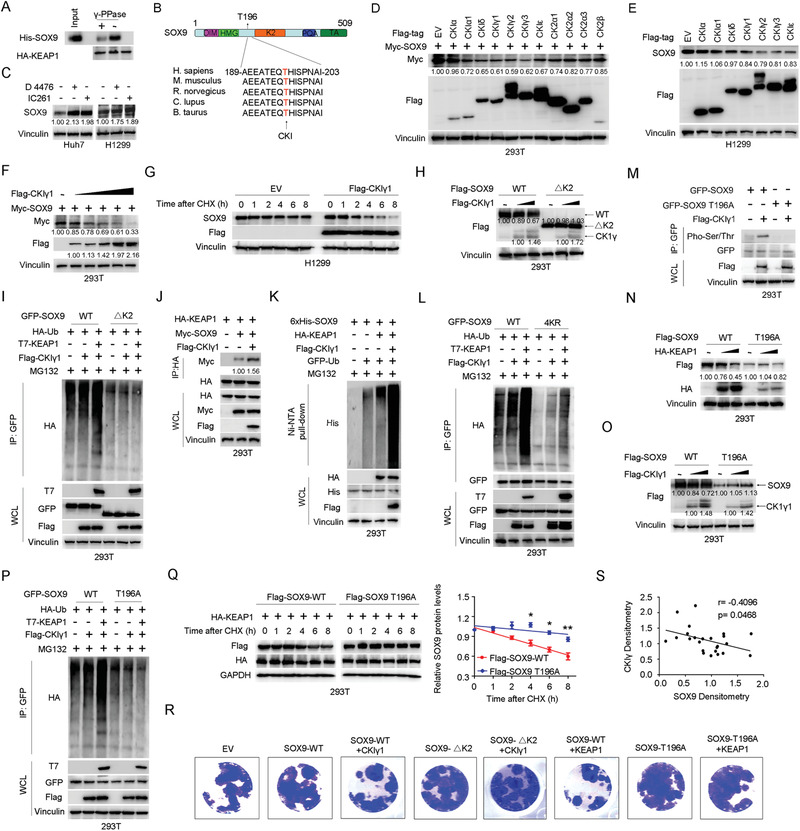
Casein kinase CKI*γ*1 phosphorylates SOX9 at T196 to trigger KEAP1 binding and subsequent SOX9 ubiquitination and degradation. A) Co‐IP analysis of KEAP1/SOX9 interaction in HEK293T cells with or without *γ* protein phosphatase (*γ*‐PPase) treatment. The treatment was performed for 1 h at 37 °C prior to HA‐KEAP1 IP and His‐SOX9 IB. B) Sequence alignment of the amino acid 189–203 region in SOX9 harboring the T196 phosphorylation site across various species. C) Immunoblotting (IB) analysis of SOX9 protein levels in Huh7 and H1299 cells treated with the CKI inhibitors IC261 (50 × 10^−6^ m) or D4476 (20 × 10^−6^ m) for 10 h before harvesting. D,E) IB analysis of SOX9 protein levels in HEK293T D) and H1299 E) cells expressing the indicated casein kinases. F) IB analysis of SOX9 protein levels in HEK293T cells expressing increasing amount of Flag‐CKI*γ*1. G) Protein half‐life analysis of SOX9 in H1299 cells expressing Flag‐CKI*γ*1. A total of 36 h after plasmid transfection, cells were treated with 10 µg mL^−1^ cycloheximide (CHX) for the indicated time period before they were harvested for IB analyses. H) IB analysis of the protein levels of wild‐type (WT) SOX9 and the SOX9 K2 domain deletion mutant (ΔK2) in HEK293T cells expressing increasing amounts of Flag‐CKI*γ*1. I) In vivo ubiquitination assay of GFP‐tagged WT‐SOX9 and the SOX9 ΔK2 mutant in HEK293T cells with or without ectopic CKI*γ*1 and KEAP1 expression. A total of 36 h after plasmid transfection, cells were treated with 20 × 10^−6^ m MG132 for 6 h before harvesting. J) Co‐IP analysis of KEAP1/SOX9 interaction in HEK293T cells with or without ectopic CKI*γ*1 expression. K) In vivo ubiquitination assay of SOX9 in HEK293T cells with or without ectopic CKI*γ*1 and KEAP1 expression. L) In vivo ubiquitination assay of GFP‐tagged WT‐SOX9 and the SOX9 4KR mutant in HEK293T cells with or without ectopic CKI*γ*1 and KEAP1 expression. M) In vivo phosphorylation assay of GFP‐tagged WT‐SOX9 and the SOX9 T196A mutant in HEK293T cells with or without ectopic expression of Flag‐CKI*γ*1. N,O) IB analysis of WT‐SOX9 and the SOX9 T196A mutant protein levels in HEK293T cells expressing increasing amounts of HA‐KEAP1 N) or Flag‐CKI*γ*1 O). P) In vivo ubiquitination assay of GFP‐tagged WT‐SOX9 and the SOX9 T196A mutant in HEK293T cells with or without ectopic CKI*γ*1 and KEAP1 expression. Q) Protein half‐life analysis of Flag‐tagged WT‐SOX9 and SOX9 T196A mutant in H1299 cells expressing HA‐KEAP1. Quantification of SOX9 levels relative to GAPDH was shown. Data are presented as mean ± SEM. *n* = 3 independent experiments. ^:^ *p* < 0.05, ^::^ *p* < 0.01, Student's *t* test. R) Representative images from colony formation assays of H1299 cells stably expressing WT‐SOX9 and the indicated SOX9 mutants in the presence or absence of ectopic CKI*γ*1 and KEAP1 expression. S) Statistical analysis of the correlation between SOX9 and CKI*γ* protein levels in lung carcinoma patients.

Although proper substrate phosphorylation is required for recognition by many well‐studied SCF‐type E3 ubiquitin ligases, such as FBW7 and *β*‐TRCP,^[^ ^11^, ^37^ ^]^ it is unclear whether similar mechanism also apply for the Cullin 3‐based KEAP1 E3 ligase to recognize its substrates. Intriguingly, we noticed that the interaction between SOX9 and KEAP1 was reduced significantly upon *γ* protein phosphatase (*γ*‐PPase) treatment (**Figure** **7**A), suggesting that the phosphorylation of SOX9 facilitates its interaction with KEAP1. Threonine 236 (T236), but not T240, of SOX9 is phosphorylated by GSK3 kinase, consequently resulting in SOX9 degradation by SCF^FBW7*α*^.^[^ ^11^ ^]^ However, mutating SOX9 T236 and T240 was unable to inhibit KEAP1‐mediated SOX9 degradation (Figure S5A, Supporting Information), suggesting the existence of other functional phosphorylation sites responsible for mediating KEAP1‐SOX9 interaction. To look for these functional phosphorylation sites, we first attempted to identify the kinase(s) responsible for SOX9 phosphorylation. The Scansite program (http://scansite.mit.edu) predicted that Ser/Thr residues of the SOX9 K2 domain linker region are likely casein kinase I (CKI) target sites (Figure 7B). As expected, we found that treatment with the CKI inhibitors IC261 or D4476, but not the CKII inhibitor CX4945, substantially prolonged the half‐life of SOX9, resulting in the accumulation of SOX9 protein (Figure 7C and Figure S5B−D, Supporting Information). Interestingly, CKI*γ*, and to a lesser extent the other CKI and CKII isoforms, could significant promote endogenous and exogenous SOX9 degradation (Figure 7D−F and Figure S5E, Supporting Information). We also observed that the half‐life of SOX9 protein was shortened in the presence of ectopic CKI*γ*1 expression (Figure 7G and Figure S5F, Supporting Information). Importantly, we further demonstrated that deletion of the K2 domain containing its linker region (182‐303 aa) largely abolished CKI*γ*1‐mediated poly‐ubiquitination and degradation of SOX9 (Figure 7H,I).

Notably, overexpression of CKI*γ*1 strongly enhanced the binding between SOX9 and KEAP1 (Figure 7J), and promoted SOX9 ubiquitination (Figure 7J,K). Importantly, mutations at the ubiquitination sites of SOX9 significantly increased resistance to CKI*γ*1‐mediated SOX9 poly‐ubiquitination (Figure 7L). To look for the major CK*γ*1‐mediated phosphorylation sites in the SOX9 K2 domain linker region, we focused on an evolutionarily conserved Thr residue T196 whose phosphorylation was detected by high‐resolution proteomic discovery mass spectrometry analysis.^[^ ^38^ ^]^ Intriguingly, point mutation of T196 residue to alanine (A) alone was sufficient to completely block CKI*γ*1‐mediated SOX9 phosphorylation (Figure 7M). Consistently, the SOX9‐T196A mutant exhibited a marked elevation in resistance to KEAP1‐ or CKI*γ*1‐mediated SOX9 poly‐ubiquitination and degradation (Figure 7N−P). As a result, exogenous SOX9‐T196A mutant displayed a significantly prolonged half‐life (Figure 7Q). Moreover, ectopic expression of KEAP1 or CKI*γ*1 could efficiently suppress cell colony formation induced by overexpression of SOX9‐WT but not SOX9ΔK2 domain or SOX9‐T196A mutant (Figure 7R). Finally, we investigated the clinical relevance of our findings using tissues obtained from lung carcinoma patients. We observed that CKI*γ* protein expression levels were negatively correlated with SOX9 protein expression levels (Figure 7S). These results coherently suggest that CK*γ*1 functions as the upstream kinase that phosphorylates the T196 residue within SOX9 K2 domain linker region, subsequently enhancing the KEAP1‐mediated interaction and degradation of SOX9 to govern its biological functions.

### 2.6 Etoposide Promotes the Degradation of SOX9 in a KEAP1‐ and CKI*γ*‐Dependent Manner

**Figure 8:**
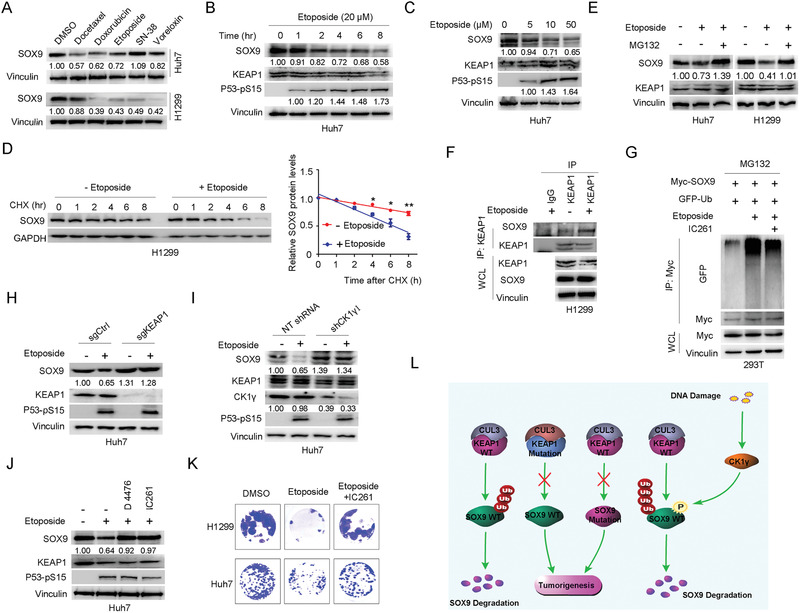
Etoposide‐induced SOX9 degradation is dependent on KEAP1 and CKI*γ*. A) Immunoblotting (IB) analysis of SOX9 protein levels in Huh7 and H1299 cells treated with 10 × 10^−6^ m of various DNA‐damaging drugs for 12 h. B,C) IB analysis of SOX9 protein levels in Huh7 cells treated with 20 × 10^−6^ m etoposide for increasing amount of time B) or increasing concentration of etoposide for 12 h C) before harvesting. D) Protein half‐life analysis of SOX9 in H1299 cells treated with or without 20 × 10^−6^ m etoposide for 12 h. Data are presented as mean ± SEM. *n* = 3 independent experiments.^:^ *p* < 0.05, ^::^ *p* < 0.01, Student's *t* test. E) IB analysis of SOX9 protein levels in Huh7 and H1299 cells treated with or without etoposide (20 × 10^−6^ m) and MG132 (20 × 10^−6^ m) for 6 h before harvesting. F) Co‐IP analysis of KEAP1/SOX9 interaction in H1299 cells with or without etoposide (20 × 10^−6^ m) treatment. G) In vivo ubiquitination assay of Myc‐tagged SOX9 in HEK293T cells with or without etoposide (20 × 10^−6^ m) and IC261 (50 × 10^−6^ m) treatment. H,I) IB analysis of SOX9 protein levels in Huh7 cells with or without etoposide treatment in the presence or absence of KEAP1 H) or CKI*γ*1 I) depletion by CRISPR/Cas9 or shRNA, respectively. P53‐pS15 was used as an indicator of DNA damage to validate the effect of etoposide treatment. J) IB analysis of SOX9 protein levels in Huh7 cells treated with etoposide (20 × 10^−6^ m) and the CKI inhibitors IC261 (50 × 10^−6^ m) and D4476 (20 × 10^−6^ m) for 8 h before harvesting. K) Representative images from colony formation assays of H1299 and Huh7 cells treated with etoposide (20 × 10^−6^ m) or etoposide plus the CKI inhibitor IC261 (50 × 10^−6^ m). L) A proposed working model of KEAP1 negatively regulates SOX9 to suppress tumorigenesis.

Due to the lack of CKI agonist, we next explored whether DNA‐damaging reagent which have been reported to activate CKI,^[^ ^39^ ^]^ could also promote KEAP1‐mediated SOX9 degradation. Indeed, we found that DNA‐damaging drugs, mainly topoisomerase inhibitors, including etoposide and doxorubicin, could significantly reduce the protein levels of SOX9 (**Figure** **8**A and Figure S6A, Supporting Information). We chose to focus on etoposide to further study how DNA damage response might govern SOX9 stability in the reminder of the study. Notably, we found that etoposide downregulated SOX9 protein levels in both time‐ and dose‐dependent manner (Figure 8B,C and Figure S6B, Supporting Information), largely by shortening the SOX9 protein half‐life (Figure 8D and Figure S6C, Supporting Information). More importantly, the etoposide‐induced SOX9 reduction could be blocked by MG132 (Figure 8E), indicating that etoposide regulates SOX9 protein levels largely in a ubiquitination‐dependent posttranslational manner. Consistently, we found that etoposide treatment resulted in an enhanced association between SOX9 and endogenous or exogenous KEAP1 (Figure 8F and Figure S6D, Supporting Information), leading to the elevated ubiquitination of SOX9 that can be prevented by treatment with the CKI inhibitor IC261 (Figure 8G). In addition, we also found that the etoposide‐induced SOX9 phosphorylation was largely abolished by T196A point mutation (Figure S6E, Supporting Information).

Consistent with our finding that KEAP1 is an E3 ubiquitin ligase that controls SOX9 stability, we found that the depletion of endogenous KEAP1 or ectopic expression of the KEAP1 mutants, R320Q and G364S could largely abolish etoposide‐triggered degradation of SOX9 in vivo and in vitro (Figure 8H and Figure S6F, Supporting Information). Consistently, inactivation of CKI*γ* by either its depletion (Figure 8I) or the use of CKI inhibitors IC261 and D4476 (Figure 8J and Figure S6G, Supporting Information) efficiently blocked etoposide‐induced SOX9 degradation. Moreover, the ability of colony formation for H1299 and Huh7 cells decreased dramatically upon etoposide treatment, and this ability was significantly restored by CKI inhibitor IC261 treatment (Figure 8K), illustrating a critical physiological role for KEAP1 in mediating the etoposide‐induced degradation of SOX9. Together, these results suggest that etoposide can suppress cancer cell proliferation by promoting the degradation of SOX9 in a KEAP1‐ and CKI*γ*‐dependent manner.

## 3 Discussion

In the current study, we provide experimental evidences demonstrating that the E3 ubiquitin ligase KEAP1 plays a critical role in oncoprotein SOX9 poly‐ubiquitination and subsequent degradation. Although the protein−protein interaction between KEAP1 and SOX9 was not reported before, we have explicitly showed in our manuscript that KEAP1 directly binds to SOX9 through its Kelch domain by serials of deletion in KEAP1 and reciprocal immunoprecipitation using antibodies against the tags and the target proteins (Figures 2 and 4). Through our studies, a subset of cancer‐associated KEAP1 mutations was found to disrupt the substrate‐recruiting function of Kelch domain to impair the ability of KEAP1 to bind and promote SOX9 poly‐ubiquitination and degradation. Therefore, these results suggest that KEAP1 mutations might favor tumorigenesis in part by enhancing the stability of the oncoprotein SOX9, at least in the lung carcinoma and HCC setting (Figure 8L).

SOX9 has emerged as a potential therapeutic target for various types of cancer.^[^ ^6^ ^]^ We and others initially identified that SOX9 is one of the key factors critical for maintaining the self‐renewal and pluripotency of tumor‐initiating cells or cancer stem cells (CSCs) in HCC and lung carcinoma.^[^ ^12^, ^14^, ^40^ ^]^ Although previous studies indicate that FBW7 regulates SOX9 protein levels by promoting SOX9 ubiquitination and degradation,^[^ ^10^, ^11^ ^]^ our work provides evidence to support that KEAP1 can also directly control SOX9 stability in a posttranslational manner. Specifically, mutations in the E3 ubiquitin ligase KEAP1 disrupt its interaction with SOX9, prevent SOX9 poly‐ubiquitination, and stabilize the protein to promote tumorigenesis. By analyzing the clinical datasets, we found that KEAP1 mutations showed significant positive correlation with SOX9 expression in the TCGA lung cancer cohort. Moreover, human KEAP1‐mutant transcriptional signature and core SOX9 target genes were significantly enriched in advanced‐stage tumors. Thus, our current study provides a potential molecular mechanism to explain the pathological increase in SOX9 stability. This occurs at least in part through the evasion of KEAP1‐mediated degradation. However, it will be important to further validate that these KEAP1 mutations are enriched in tumor‐initiating cells or CSCs.

Furthermore, both low‐affinity (DLG) and high‐affinity (ETGE) motif binding are required for KEAP1‐mediated substrate degradation, such as in the case of NRF2 and IKK*β*.^[^ ^41^, ^42^ ^]^ Ge et al. showed that the antioxidative factor iASPP drives cancer growth and drug resistance by competing with NRF2 for KEAP1 binding via DLT.^[^ ^43^ ^]^ Interestingly, SOX9 contains a DLK motif that resembles DLG in NRF2 or DLT in iASSP, but has no ETGE‐like motif. These observations imply that SOX9 binds KEAP1 via DLK, which is a novel DLG motif. Indeed, KEAP1 through the consensus binding motif ^251^DLK^253^, binds the K2 transactivation domain of SOX9, a region that has been demonstrated to play a critical role in SOX9 stability and regulation.^[^ ^11^ ^]^ As the result, the SOX9 ^251^DLK^253^ mutant is deficient in KEAP1‐binding, enabling it to evade KEAP1‐mediated degradation, thereby making it more oncogenic than the WT‐KEAP1. We also investigated whether somatic mutations exist in the SOX9 DLK region, which could prevent its binding to KEAP1 and lead to increased SOX9 protein levels. In the COSMIC dataset, we did not find any somatic mutations in this region, suggesting that dysregulation of KEAP1‐mediated SOX9 degradation resulted primarily from deficiency in the KEAP1‐CUL3 ubiquitin complex caused by KEAP1 somatic mutations or allelic loss.

Moreover, it is interesting to note that SOX9 interacts with MAF proteins, which can form heterodimers with NRF2 for transcriptional regulation.^[^ ^44^, ^45^ ^]^ NRF2 is a major target of KEAP1‐mediated degradation, and the potential cross‐talk between SOX9 and NRF2 through MAF proteins could add another layer to the sophisticated regulation of tumorigenesis process.

Although previous reports showed that the depletion of SOX9 could be induced by DNA damage through increases in its poly‐ubiquitination and degradation, the physiological upstream regulator of SOX9 stability was unknown. Here, our studies identified that the CKI*γ*1 is one such regulator, which facilitates SOX9‐KEAP1 interaction and promotes SOX9 degradation to inhibit tumorigenesis. Accordingly, CKI agonists or DNA damage drugs could inhibit SOX9‐mediated tumorigenesis by promoting KEAP1‐mediated degradation of SOX9.

In summary, we identify KEAP1 as an E3 ubiquitin ligase of SOX9. Moreover, our results indicate that a subset of cancer‐associated KEAP1 mutations abrogate this function. We also show that the K2 transactivation domain of SOX9 contains binding degron motif that is necessary for KEAP1 interaction. Although it is clear that SOX9 expression can be regulated at the transcriptional level, our data indicate that the posttranslational modulation of SOX9 stability through KEAP1‐mediated proteasome degradation represents another essential mechanism that contributes to SOX9 regulation in human lung carcinoma and HCC. Given that a subset of KEAP1 mutations lead to elevated SOX9 protein levels and tumorigenesis, our findings suggest that targeting SOX9 in cancer patients bearing such mutations could be a viable stratified approach for their treatment.

## 4 Experimental Section

##### Cell Culture, Transfection, and Viral Infections

HEK293T and human liver cancer cell lines including Huh7, PLC/PRF/5, Hep3B cells, and Huh7 derived cell lines (KEAP1‐knockout cells) were cultured in Dulbecco's modified Eagle medium (DMEM) medium (Gibco); H1299 cells were cultured in Roswell Park Memorial Institute (RPMI‐1640) medium (Gibco); A549 cells were cultured in F‐12 medium (Gibco) supplemented with 10% fetal bovine serum (FBS; Gibco), 100 units of penicillin and 100 mg mL^−1^ streptomycin in a sterile 37 °C incubator with a humidified 5% CO_2_ atmosphere. Cells were authenticated by short tandem repeat profiling. Immediately upon receipt, cells were expanded and then frozen to be revived every 3 to 4 months. All cell lines were monitored by mycoplasma PCR testing and maintained in mycoplasma‐free conditions.

Cells were transfected with various plasmids using Neofect DNA transfection reagent (Neofect) or effectene transfection reagent (QIAGEN) according to the manufacturer's protocol. Lentiviral expression and shRNA virus packaging and subsequent infection of various cell lines were performed according to the protocol described previously.^[^ ^46^ ^]^ For lentivirus‐mediated gene knockdown or overexpression experiments, Huh7 and H11299 cells were infected with the same virus multiplicity of infection. After overnight incubation, the medium was refreshed. Puromycin was then added for selection for 72 h at 1 µg mL^−1^ final concentration. After confirming the gene knockdown and overexpression efficiency by western blotting, colony formation and tumor growth from these cells were assessed as described previously.^[^ ^46^ ^]^

##### CRISPR/Cas9‐Mediated Deletion of KEAP1

KEAP1‐KO cell lines were generated using the *Keap1* gene‐specific CRISPR/Cas9/GFP plasmids (sc‐400190‐KO‐2; Santa Cruz). This CRISPR/Cas9 KO product consists of a mixture of three plasmids each encoding a Cas9 nuclease and a *Keap1* gene‐specific 20‐nt guide (g)RNA designed for maximum KO efficiency. These gRNA sequences were derived from the GeCKO (v2) library and they specifically direct the Cas9 nuclease to target gene to induce site‐specific double strand break in the genomic DNA. Huh7 cells were transfected with the above‐mentioned plasmids then sorted with GFP by FACS. Single cell cloning was performed by serial dilution of the sorted cells in 96‐well plate, followed by immunoblotting analysis of KEAP1 to select the clones with complete gene knockout.

##### Drug Treatments

Drugs used in the study include: Bortezomib (S1013), MLN4924 (S7109), MG132 (S2619), Docetaxel (S1148), Doxorubicin (S1208), Etoposide (S1255), SN‐38 (S4908), and Voreloxin (S7518) from Selleckchem. Cycloheximide (CHX; N11534) was purchased from Sigma‐Aldrich. Drugs were dissolved in dimethyl sulfoxide (DMSO). Prior to drug treatment, cells were plated in six‐well plates. When cells reached 60% confluence, they were treated with indicated drugs for various length of time at the concentration described in corresponding figure legend. After treatment, cells were collected for protein extraction and immunoblotting analysis.

##### Protein Half‐Life Assays

Cells with specific gene depletion or overexpression or drug treatment were assayed for SOX9 protein stability with appropriate controls. To determine the half‐life of SOX9, we applied the classic CHX chase assay. CHX is an inhibitor of protein biosynthesis and its treatment allows for the accurate determination of the degradation rate of existing proteins. In the SOX9 half‐life assays, CHX (10 µg mL^−1^) was added to the cell culture and at the indicated time points thereafter, cells were harvested and protein abundances were measured by immunoblotting analysis.

##### In vitro Ubiquitination Assay

In vitro ubiquitination assay was carried out as described previously.^[^ ^47^ ^]^ Briefly, HEK293T cells were transfected with plasmids expressing Flag‐KEAP1, Myc‐Cullin 3, and HA‐RBX1 to purify KEAP1/Cullin 3/RBX1 complex by Flag affinity precipitation. 6× His‐SOX9 protein was purified by nickel‐nitrilotriacetic acid (Ni‐NTA) matrices (QIAGEN). The ubiquitination assay was carried out at 37 °C for 2 h in 20 µL reaction buffer (20 × 10^−3^ m Tris‐HCl, pH 7.2; 5 × 10^−3^ m MgCl_2_; 50 × 10^−3^ m NaCl; 1 × 10^−3^ m 2‐mercaptoethanol; 10% glycerol) containing the following components: 100 × 10^−9^ m UBE1, 2 × 10^−6^ m UbcH5a, 4 × 10^−3^ m ATP, 1 × 10^−6^ m ubiquitin aldehyde, 50 × 10^−6^ m ubiquitin WT or Lys 6 only, or Lys 11 only, or Lys 27 only, or Lys 29 only, or Lys 33 only, or Lys 48 only, or Lys 63 only (all from Ubiquitin‐Proteasome Biotechnologies, Cat# J3220), 5 × 10^−6^ m of Flag‐KEAP1/Cullin 3/RBX1 complex and His‐SOX9. The reaction was terminated by adding 0.4 mL pulldown buffer (20 × 10^−3^ m Tris‐HCl, pH 7.5; 500 × 10^−3^ m NaCl; 1% Triton X‐100; 0.02% BSA; and 5 × 10^−3^ m *β*‐mercaptoethanol). After the addition of 5 µL His‐tag antibody or SOX9 antibody, the samples were rotated at 4 °C for 6 h. The primary antibodies were then pulled down with 60 µL Protein G sepharose beads (Cat# 88 848, Thermo Fisher Scientific) by incubating for 6 h at 4 °C. The beads were washed with 1 mL of pulldown buffer three times. The proteins bound to beads were released by boiling in 50 µL of 2× SDS‐PAGE sample buffer for 10 min. The samples were then resolved by 8% SDS‐PAGE followed by immunoblot analysis.

##### Mouse Xenograft Assays

All NOD/SCID mice used in this research were obtained from the Army Medical University and maintained in pathogen‐free conditions. The procedures related to animal studies were approved by the Ethics Committee of the Institutional Review Board of the Southwest Hospital, Army Medical University and conformed to the NIH guidelines on the ethical use of animals. The sample sizes of the animals were justified by statistical considerations and statistical power analyses. The animals were allocated for different experiments and outcome assessments randomly. H1299 (5 × 10^6^) or Huh7 (1 × 10^6^) cells in a volume of 100 µL PBS were injected subcutaneously into both flanks of 4−5‐week‐old male NOD/SCID mice as described previously.^[^ ^46^ ^]^ At the end of the experiments, the mice were humanely killed, and tumor of each mouse was harvested, then tumor weights were measured and recorded postnecropsy. Tumor size was measured every 2 days with a caliper, and the tumor volumes were calculated using the formula *V* = (*π*/6) × *a* × *b* ^2^, where *a* and *b* are the long axis and short axis of tumor, respectively.

##### SOX9 and KEAP1‐Mutant Target Gene Sets Analyses

An initial set of SOX9 target genes was extracted from SOX9 binding motifs from the JASPAR Predicted Transcription Factor Targets dataset (http://amp.pharm.mssm.edu/Harmonizome/gene_set/SOX9/JASPAR+Predicted+Transcription+Factor+Targets). To determine a cell‐type specific list of SOX9 target genes for lung cancer, we performed differential gene expression analysis on tumors with matched normal samples in the respective TCGA datasets. The raw RNA‐seq read counts were used in the DESeq2 pipeline to determine the differential gene expression of the genes (with predicted SOX9 binding sites) in the tumors relative to the paired normal samples.^[^ ^48^, ^49^ ^]^ A list of significantly (adjusted *p*‐value ≤ 0.05) differentially expressed SOX9 target genes used to calculate the SOX9 gene scores in lung cancer is available in Table S1, Supporting Information.

Similarly, expression profiles from the TCGA human lung cancer cohort were analyzed to derive a KEAP1‐mutant gene expression signature. Using mutation cells from TCGA, primary tumor samples from subjects with protein‐altering mutations in KEAP1 (*n* = 40) and WT‐KEAP1 (*n* = 468) were identified. Independent Component Analysis (ICA) was performed on the combined dataset to categorize the genes by the gene expression profiles using the Joint Approximate Diagonalization of Eigenmatrices (JADE) algorithm in R.^[^ ^23^, ^50^ ^]^ The gene expression profiles of the individual resultant components (gene sets) were then evaluated for the ability to distinguish between the KEAP1 mutant and WT tumors using a Mann−Whitney‐Wilcoxon test. The genes from the resultant statistically significant expression profiles with a |z‐score| > 2 were used to form the gene sets (upregulated gene set and downregulated gene set) for subsequent computation of the KEAP1‐mutant gene score. A list of the gene sets that were used in the KEAP1 mutant gene score calculation is available in Table S2, Supporting Information.

##### SOX9 and KEAP1‐Mutant Core Target Signature

The gene expression profiles (normalized counts) for individual primary tumor samples were scored with the gene expression signatures (SOX9 or KEAP1‐mutant target gene sets) using ssGSEA from the GSVA package (v1.32) in R.^[^ ^51^, ^52^, ^53^ ^]^ This was performed separately for the upregulated and downregulated genes. The final gene score was determined by subtracting the downregulated gene score from the upregulated gene score. Once the scores were calculated, the patients can then be stratified into top‐scoring percentile versus the rest of the cohort. To determine the KEAP1‐mutant gene expression profile in tumors expressing high SOX9 target gene expression (Figure 5I), we stratified the tumors into SOX9 high (top 10 percentile in terms of SOX9 gene score) relative to SOX9 low (rest of the tumors). Additionally, the lung cancer tumors were stratified into KEAP1 (mutant/nonmutant) and SOX9 (high/low) groups to perform Kaplan−Meier survival analyses using overall survival time, with the log‐rank test used to determine the statistical significance (Figure 5L). For the survival analysis, the median (50^th^ percentile) SOX9 gene score was used as the threshold. Tumors with a larger SOX9 gene score than this threshold were categorized as “SOX9 high” while the tumors with lower SOX9 gene score were categorized as “SOX9 low”. A more lenient definition of the term “SOX9 high” was necessary due to the relatively small number of KEAP1 mutant tumors compared to the KEAP1 WT tumors. A list of the SOX9 and KEAP1‐mutant gene scores with the clinical and mutation data is available in Table S3, Supporting Information.

Results pertaining to the distribution of gene scores for each cancer type were visualized with empirical cumulative distribution function (ECDF) plots with Kolmogorov−Smirnov test applied to assess statistical significance between different ECDFs. All statistical analyses were performed in R (https://www.r-project.org/).

##### Statistical Analysis

Results are reported as mean ± standard error of the mean (SEM) of multiple independent experiments, not technical replicates. Differences between variables were assessed by two‐tailed Student's *t* test, one‐way ANOVA, and *χ* ^2^ test, where appropriate. All statistical analyses tests were performed with GraphPad Prism 5.0 and SPSS19.0 software. Values with *p* < 0.05 were considered statistically significant, with ^:^ *p* < 0.05, ^::^ *p* < 0.001, ^:::^ *p* < 0.0001, unless otherwise indicated in the figure.

## Conflict of Interest

The authors declare no conflict of interest.

## Author Contributions

N.S. and C.L. designed and performed most of the experiments. H.H. constructed the plasmids and assisted the experiments. N.S., M.I. and X.P. provided the administrative, technical, and material support. C.L analyzed the data and provided funds. F.X. read and wrote the manuscript. C.L. and S.D. conceived the project, supervised the study, and wrote the manuscript. All authors commented on the manuscript.

## Supporting information

Supporting Information
