# Supplementary material for: KEAP1 Mutations Drive Tumorigenesis by Suppressing SOX9 Ubiquitination and Degradation
Source: Adv Sci (Weinh). 2020 Sep 27;7(21):2001018. doi: 10.1002/advs.202001018 (PMC7610265; doi:10.1002/advs.202001018)
Supplement: Supplementary file 1 — Supporting Information [file ADVS-7-2001018-s001.pdf]

© 2020 Wiley-VCH GmbH

Supporting Information

for *Adv. Sci.,* DOI: 10.1002/advs.202001018

KEAP1 MutationsDrive Tumorigenesis by Suppressing SOX9 Ubiquitination and Degradation

Na Shao, Hong Huang, Muhammad Idris, Xu Peng, Feng Xu*, Shiwu Dong*, Chungang Liu*

Supporting Information

KEAP1 Mutations Drive Tumorigenesis by Suppressing SOX9 Ubiquitination and Degradation

Na Shao[1], Hong Huang[2], Muhammad Idris[3], Xu Peng[3], Feng Xu[3*], Shiwu Dong[1*], Chungang Liu[2, 3*]

[1]Department of Biomedical Materials Science, School of Biomedical Engineering, Army Medical University, Chongqing 400038, P.R. China

[2]Center of Biological Therapy, Southwest Hospital, Army Medical University, Chongqing 400038, P.R. China

[3]Institute of Molecular and Cell Biology, Agency for Science, Technology and Research (A*STAR), Singapore

Contents of supporting information

Supporting Figures 1-6

Extended experimental procedures

Supporting Tables 1-3

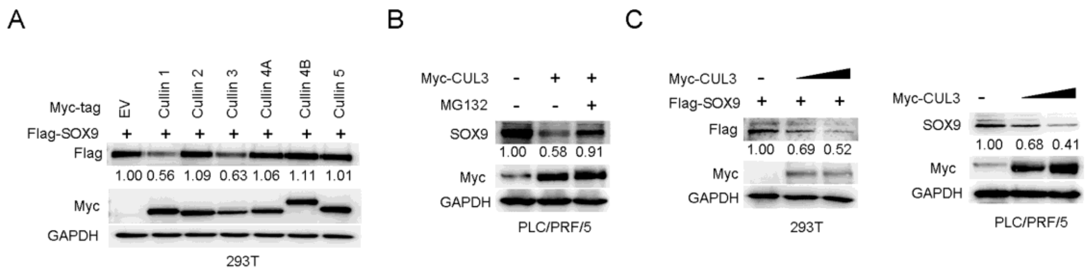
A
Myc-tag
EV
Cullin 1
Cullin 2
Cullin 3
Cullin 4A
Cullin 4B
Cullin 5
Flag-SOX9
Flag
1.00 0.56 1.09 0.63 1.06 1.11 1.01
Myc
GAPDH
293T
B
Myc-CUL3
MG132
SOX9
1.00 0.58 0.91
Myc
GAPDH
PLC/PRF/5
C
Myc-CUL3
Flag-SOX9
Flag
1.00 0.69 0.52
Myc
GAPDH
293T
Myc-CUL3
SOX9
1.00 0.68 0.41
Myc
GAPDH
PLC/PRF/5

Figure S1

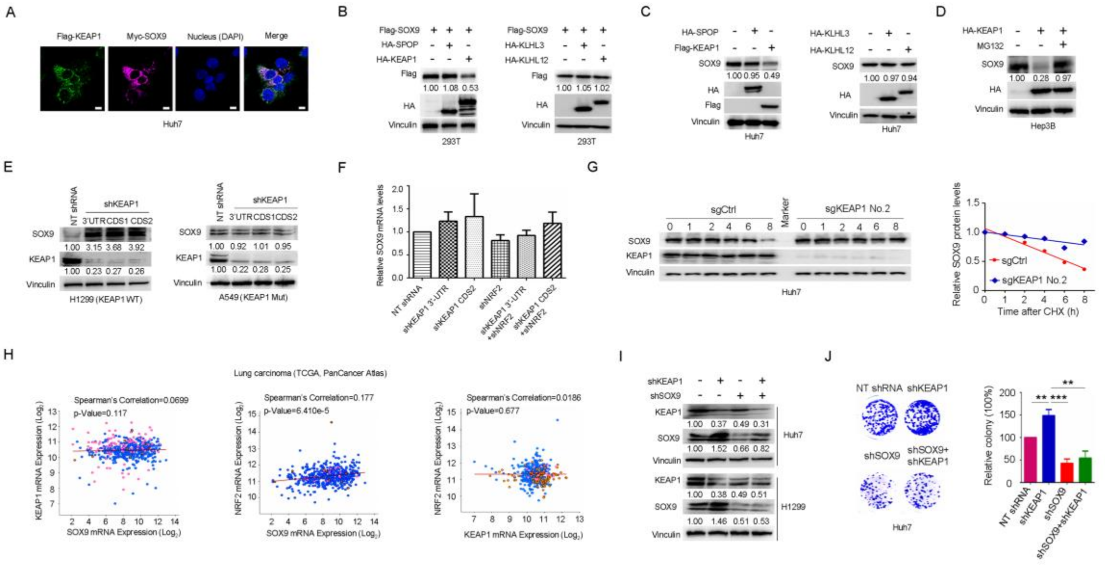

Figure S2

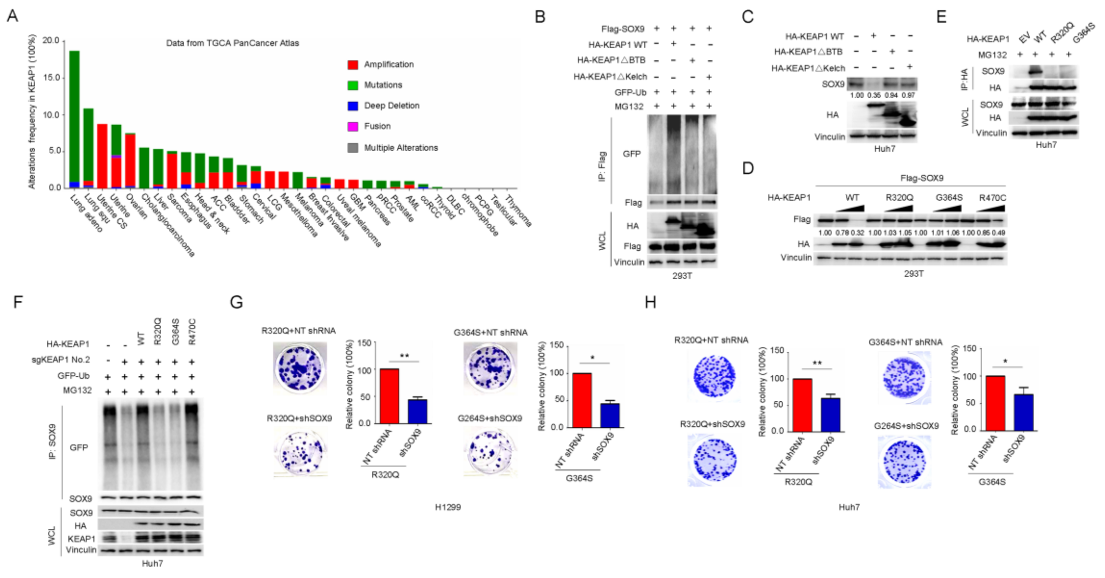
A
Data from TGCA PanCancer Atlas
Alterations frequency in KEAP1 (100%)
Amplification
Mutations
Deep Deletion
Fusion
Multiple Alterations
B
C
D
E
F
G
H
H1299
Huh7
293T

Figure S3

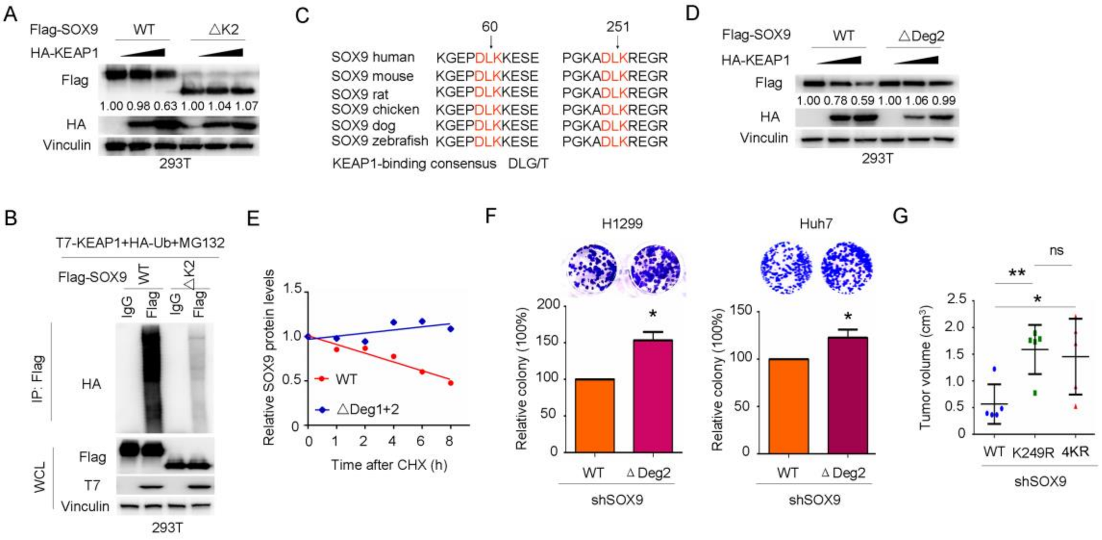
A
Flag-SOX9 WT △K2
HA-KEAP1
Flag
1.00 0.98 0.63 1.00 1.04 1.07
HA
Vinculin
293T
B
T7-KEAP1+HA-Ub+MG132
Flag-SOX9 WT △K2
IgG Flag IgG Flag
IP: Flag
HA
WCL
Flag
T7
Vinculin
293T
C
60
251
SOX9 human KGEPDLKKESE PGKADLKREGR
SOX9 mouse KGEPDLKKESE PGKADLKREGR
SOX9 rat KGEPDLKKESE PGKADLKREGR
SOX9 chicken KGEPDLKKESE PGKADLKREGR
SOX9 dog KGEPDLKKESE PGKADLKREGR
SOX9 zebrafish KGEPDLKKESE PGKADLKREGR
KEAP1-binding consensus DLG/T
D
Flag-SOX9 WT △Deg2
HA-KEAP1
Flag
1.00 0.78 0.59 1.00 1.06 0.99
HA
Vinculin
293T
E
Relative SOX9 protein levels
WT
△Deg1+2
Time after CHX (h)
F
H1299
Huh7
Relative colony (100%)
WT △Deg2
shSOX9
G
Tumor volume (cm³)
ns
**
*
WT K249R 4KR
shSOX9

Figure S4

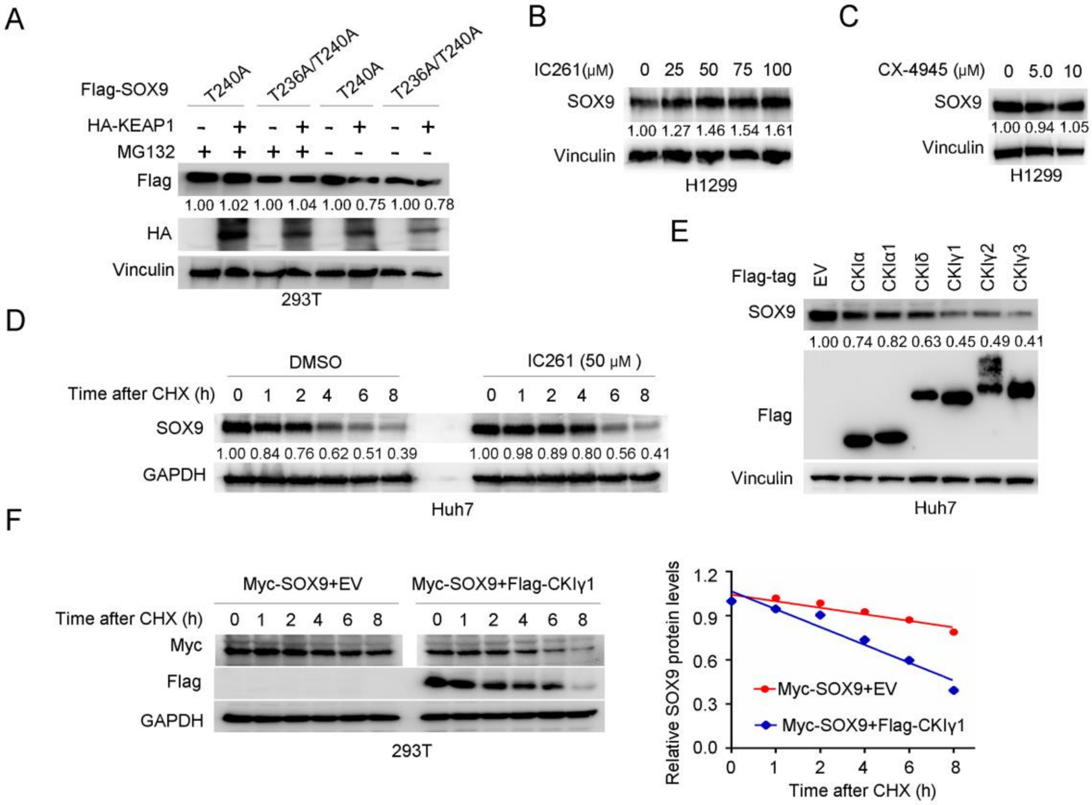
A
Flag-SOX9
T240A
T236A/T240A
T240A
T236A/T240A
HA-KEAP1
MG132
Flag
1.00 1.02 1.00 1.04 1.00 0.75 1.00 0.78
HA
Vinculin
293T
B
IC261(μM) 0 25 50 75 100
SOX9
1.00 1.27 1.46 1.54 1.61
Vinculin
H1299
C
CX-4945 (μM) 0 5.0 10
SOX9
1.00 0.94 1.05
Vinculin
H1299
D
DMSO
IC261 (50 μM)
Time after CHX (h) 0 1 2 4 6 8 0 1 2 4 6 8
SOX9
1.00 0.84 0.76 0.62 0.51 0.39 1.00 0.98 0.89 0.80 0.56 0.41
GAPDH
Huh7
E
Flag-tag EV CKIα CKIα1 CKIδ CKIγ1 CKIγ2 CKIγ3
SOX9
1.00 0.74 0.82 0.63 0.45 0.49 0.41
Flag
Vinculin
Huh7
F
Myc-SOX9+EV
Myc-SOX9+Flag-CKIγ1
Time after CHX (h) 0 1 2 4 6 8 0 1 2 4 6 8
Myc
Flag
GAPDH
293T
Relative SOX9 protein levels
Time after CHX (h)
Myc-SOX9+EV
Myc-SOX9+Flag-CKIγ1

Figure S5

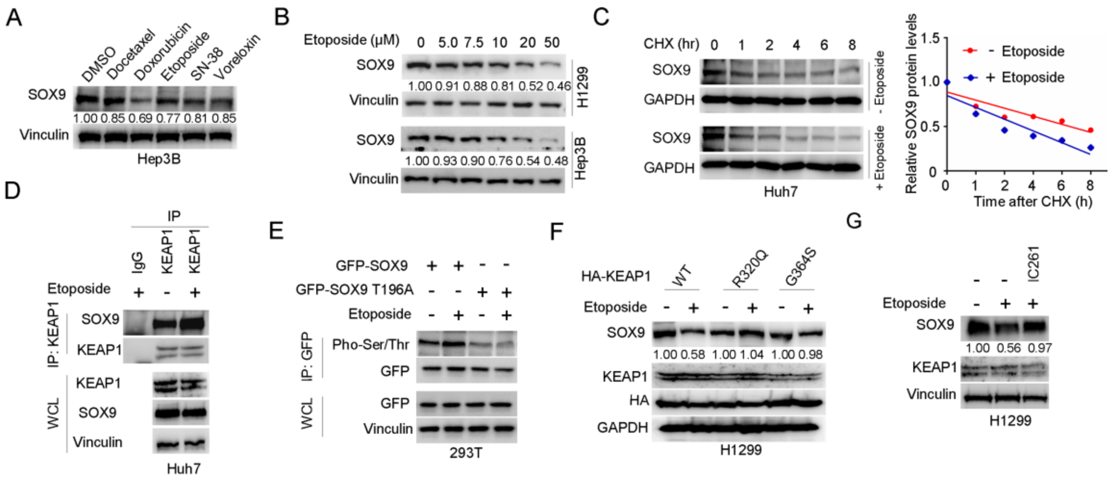
A
DMSO
Docetaxel
Doxorubicin
Etoposide
SN-38
Voreloxin
SOX9
1.00 0.85 0.69 0.77 0.81 0.85
Vinculin
Hep3B
B
Etoposide (μM) 0 5.0 7.5 10 20 50
SOX9
1.00 0.91 0.88 0.81 0.52 0.46
Vinculin
H1299
SOX9
1.00 0.93 0.90 0.76 0.54 0.48
Vinculin
Hep3B
C
CHX (hr) 0 1 2 4 6 8
SOX9
GAPDH
- Etoposide
SOX9
GAPDH
+ Etoposide
Huh7
Relative SOX9 protein levels
Time after CHX (h)
- Etoposide
+ Etoposide
D
IP
IgG
KEAP1
KEAP1
Etoposide + - +
IP: KEAP1
SOX9
KEAP1
WCL
KEAP1
SOX9
Vinculin
Huh7
E
GFP-SOX9 + + - -
GFP-SOX9 T196A - - + +
Etoposide - + - +
IP: GFP
Pho-Ser/Thr
GFP
WCL
GFP
Vinculin
293T
F
HA-KEAP1
WT
R320Q
G364S
Etoposide - + - + - +
SOX9
1.00 0.58 1.00 1.04 1.00 0.98
KEAP1
HA
GAPDH
H1299
G
IC261
Etoposide - + +
SOX9
1.00 0.56 0.97
KEAP1
Vinculin
H1299

Figure S6

Figure S1. CUL3 governs the stability of SOX9 protein.

(A) Immunoblot (IB) analysis of SOX9 protein levels in HEK293T cells transfected with Flag-tagged SOX9 (Flag-SOX9) construct plus empty vector (EV) or vectors encoding various Myc-tagged Cullin proteins.

(B) IB analysis of SOX9 protein levels in PLC/PRF/5 cells over-expressing Myc-CUL3. 36 h after plasmid transfection, cells were treated with 20 μM MG132 for 6 h before harvesting.

(C) IB analysis of SOX9 protein levels in HEK293T and PLC/PRF/5 cells transfected with Flag-SOX9 construct together with increasing amounts of Myc-CUL3 construct.

Figure S2. KEAP1 controls the stability of SOX9 protein.

(A) Myc-SOX9 was co-expressed with Flag-KEAP1 in Huh7 cells. The cellular localization of SOX9 (Magenta) and KEAP1 (Green) was examined by immunofluorescence (IF) staining (nuclei were stained with DAPI; blue). Scale bar, 10 μm.

(B, C) IB analysis of SOX9 protein levels in HEK293T cells (B) and Huh7 cells (C) transfected with indicated plasmids for 36 h before harvesting.

(D) IB analysis of SOX9 protein levels in Hep3B cells expressing HA-KEAP1 plasmid. 36 h after transfection, cells were treated with 20 μM MG132 for 6 h before they harvesting.

(E) IB analysis of SOX9 protein levels in H1299 and A549 cells infected with the indicated lentiviral KEAP1 shRNAs for 72 h before harvesting.

(F) qRT-PCR analysis of SOX9 mRNA levels from H1299 cells transfected with the indicated shRNAs for 72 h before they were harvested. Data were shown as mean ± SEM of three independent experiments.

(G) Protein half-life assay was performed for the assessment of SOX9 stability in Huh7 cells

with KEAP1 knockout. 36 h after plasmid transfection, cells were treated with cycloheximide (CHX, 10 μg/ml) for the indicated time period before they were harvested for IB analyses. Quantification of SOX9 levels relative to Vinculin was shown.

(H) Spearman's correlation analyses between SOX9, NRF2, and KEAP1 mRNA levels in The Cancer Genome Atlas (TCGA) data set of human lung carcinoma.

(I) IB analysis of KEAP1 and SOX9 protein levels in Huh7 and H1299 cells infected with the indicated lentiviral shRNAs for 72 h before harvesting.

(J) Colony-formation assay of Huh7 cells with KEAP1 and/or SOX9 shRNA knockdown. Data were presented as mean ± SEM of three independent experiments. $^{**}p<0.01$, $^{***}p<0.001$, Student's t test.

Figure S3. Cancer-associated KEAP1 mutations lead to elevated SOX9 protein abundance and cell proliferation.

(A) TCGA genome sequencing results showing that KEAP1 gene is mutated at high frequencies in a variety of human cancers, including lung cancer, liver cancer and melanoma.

(B) In *vivo* ubiquitination analysis of SOX9 in HEK293T cells expressing HA-tagged WT KEAP1 or mutant KEAP1 with BTB domain deletion (ΔBTB) or Kelch domain deletion (ΔKelch). 36 h after plasmid transfection, cells were treated with 20 μM MG132 for 6 h before they were harvested.

(C) IB analysis of SOX9 protein levels in Huh7 cells expressing HA-tagged WT KEAP1 or mutant KEAP1 ΔBTB or ΔKelch. Cells were harvested 48 h after transfection with indicated plasmids.

(D) IB analysis of SOX9 protein levels in HEK293T cells transfected with increasing amounts

of HA-tagged WT KEAP1 or cancer-associated KEAP1 mutants. Cells were harvested 48 h after transfection with indicated plasmids.

(E) Co-IP analysis of SOX9-KEAP1 interaction in Huh7 cells expressing HA-tagged WT KEAP1 or the indicated cancer-associated KEAP1 mutants. 36 h after transfection, cells were treated with 20 μM MG132 for 6 h before harvesting.

(F) In *vivo* ubiquitination analysis of SOX9 in KEAP1-KO Huh7 cells expressing WT KEAP1 or the indicated cancer-associated KEAP1 mutants. 36 h after plasmid transfection, cells were treated with 20 μM MG132 for 6 h before they were harvested.

(G and H) Colony formation assays of H1299 cells (G) and Huh7 cells (H) stably expressing KEAP1 mutants after lentiviral infection of NT shRNA or shSOX9. Data were presented as mean ± SEM of three independent experiments. *$p<0.05$, **$p<0.01$, Student's t test.

Figure S4. Deletion of degron 2 in SOX9 impairs KEAP1 binding and degradation of SOX9.

(A) IB analysis of the protein levels of WT SOX9 and SOX9 K2 domain deletion mutant (ΔK2) in HEK293T cells expressing increasing amounts of HA-KEAP1.

(B) In *vivo* ubiquitination analysis of WT SOX9 and SOX9 ΔK2 mutant in HEK293T cells expressing T7-tagged WT KEAP1. 36 h after plasmid transfection, cells were treated with 20 μM MG132 for 6 h before they were harvested.

(C) Sequence alignment of SOX9 at regions containing its KEAP1 binding motifs (degrons, highlighted in red) across various species.

(D) IB analysis of the protein levels of WT SOX9 and the SOX9 degron2 deletion mutant (ΔDeg2) in HEK293T cells expressing increasing amounts of HA-KEAP1.

(E) Quantification of the band intensities in Figure 6H.

(F) Colony formation assay of H1299 and Huh7 cells stably expressing SOX9 WT or SOX9 ΔDeg2 mutant with the endogenous SOX9 knocked down by shRNA. Data were presented as mean ± SEM of three independent experiments. *p<0.05, Student's t test.

(G) Tumor volume at the endpoint of the study was measured as indicated in Figure 6M. Data were presented as mean ± SEM, n = 5 per group. *p<0.05, **p<0.01, Student's t test.

Figure S5. The casein kinase CKIγ1 promotes SOX9 degradation.

(A) IB analysis of the indicated SOX9 mutant protein levels in HEK293T cells with or without HA-KEAP1 expression. 36 h after plasmid transfection, cells were treated with or without 20 μM MG132 for 6 h before they were harvested for further analyses.

(B, C) IB analysis of SOX9 protein levels in H1299 cells treated with increasing dose of CKI inhibitor IC261(B) or CKII inhibitor CX-4945 (C) for 10 h before harvesting.

(D) Protein half-life analysis of SOX9 in Huh7 cells treated with or without IC261 (50 uM) for 10 h before performing the analysis.

(E) IB analysis of SOX9 protein levels in Huh7 cells expressing the indicated casein kinases.

(F) Protein half-life assay was performed for the measurement of SOX9 stability in HEK293T cells expressing Flag-CKIγ1. 36 h after plasmid transfection, cells were treated with 10 μg/ml CHX for the indicated time period before they were harvested for IB analyses.

Figure S6. Etoposide-induced SOX9 degradation is dependent on KEAP1 and CKIγ.

(A) Immunoblot (IB) analysis of SOX9 protein levels in Hep3B cells treated with 10 μM of various DNA-damaging drugs for 12 h.

(B) IB analysis of SOX9 protein levels in H1299 and Hep3B cells treated with increasing

doses of etoposide for 12 h before harvesting.

(C) Protein half-life analysis of SOX9 in Huh7 cells treated with or without etoposide (20 μM) for 12 h before performing the assay.

(D) Co-IP analysis of SOX9-KEAP1 interaction in Huh7 cells with or without etoposide (20 μM) treatment.

(E) In *vivo* phosphorylation assay of GFP-tagged WT-SOX9 and the SOX9 T196A mutant in HEK293T cells treated with or without 20 μM etoposide.

(F) IB analysis of SOX9 protein levels in H1299 cells expressing WT KEAP1 or the indicated KEAP1 mutants. Cells were treated with or without 20 μM etoposide for 12 h before harvesting.

(G) IB analysis of SOX9 protein levels in H1299 cells treated with etoposide (20 μM) or etoposide (20 μM) together with the CKI inhibitor IC261 (50 μM) for 12 h before harvesting.

EXTENDED EXPERIMENTAL PROCEDURES

Antibodies

All antibodies were used at a dilution of 1:1,000 in TBST buffer with 5% non-fat milk for immunoblotting. Vinculin (V9131) and Flag (F1804) were purchased from Sigma-Aldrich. SOX9 (82630S), Cullin 3 (2759S), KEAP1 (8047S), HA-Tag (3724S), GFP-Tag (2956S), T7-Tag (13246S), Myc-Tag (2278S), His-Tag (12698S), RBX1 (11922S), Phospho-p53 (Ser15) (9286S), Ubiquitin (3936S), Phospho-(Ser/Thr)Phe (9631), NRF2 (12721S), GAPDH (2118L) were purchased from Cell Signaling Technology. KEAP1 (10503-2-AP) was purchased from Proteintech. SOX9 (sc-166505) was purchased from Santa Cruz Biotechnology, Inc. CK1γ (AF3186-SP) was purchased from R&D Systems.

Plasmids, short hairpin (sh)RNAs, and lentiviruses

Expression vectors harboring Flag-SOX9-T236A, Flag-SOX9-T236A/T240A, Myc-Cullin constructs (Cullin 1, 2, 3, 4A, 4B, and 5), GFP-Ubiquitin (Ub), T7-KEAP1, HA-KEAP1, Flag-KEAP1, HA-RBX1, HA-SPOP, HA-Ub (WT), HA-Ub (K6R), HA-Ub (K11R), HA-Ub (K27R), HA-Ub (K29R), HA-Ub (K33R), HA-Ub (K48R), HA-Ub (K63R) were purchased from Addgene. HA-KLHL2, HA-KLHL3, HA-KLHL12, HA-KLHL20, HA-KLHL37, Flag-CKI constructs (CKIα, CKIα1, CKIδ, CKIγ1, CKIγ2, CKIγ3, and CKIε) and Flag-CKII constructs (CK2α1, CK2α2, CK2α3, and CK2β) were purchased from Sino Biological Inc. Flag-KEAP1 ΔBTB and Flag-KEAP1 ΔKelch were generated by sub-cloning the corresponding cDNAs into the pcDNA3.1-Flag vector via XhoI and BamHI sites. HA-KEAP1 ΔNTR, HA-KEAP1 ΔBTB, HA-KEAP1 ΔIVR, HA-KEAP1 ΔDGR, and HA-KEAP1 ΔCTR were generated by sub-cloning the corresponding cDNAs into the pcDNA3.1-HA vector via

XhoI and BamHI sites. Flag-SOX9 construct had been described previously.[1] Myc-SOX9 and 6×His-SOX9 were constructed by cloning the corresponding cDNAs into pCMV-Myc or pCMV-6×His vector via XhoI/KpnI sites. Flag-SOX9 (aa 1-102), Flag-SOX9 (aa 103-181), Flag-SOX9 (aa 182-303), Flag-SOX9 (aa 304-379), Flag-SOX9 (aa 380-509), Flag-SOX9 ΔDLK1, Flag-SOX9 ΔDLK2, and Flag-SOX9 ΔDLK1+DLK2 were generated by sub-cloning the corresponding cDNAs into the pCMV-Flag or pCMV-Myc vector via HindIII/MluI or XhoI/KpnI sites. GFP-SOX9 (K68R), GFP-SOX9 (K82R), GFP-SOX9 (K137R), GFP-SOX9 (K249R), GFP-SOX9 (K68/82/137/249/R, 4KR), GFP-SOX9 (T196A), Flag-SOX9 (T196A), Flag-KEAP1 (R320Q), Flag-KEAP1 (G364S), and Flag-KEAP1 (R470C) mutants were generated using the Quick Change Q5 Site-Directed Mutagenesis Kit (NEBaseChanger) according to the manufacturer's instruction. HA- or 6×His-Ub (WT), HA- or 6×His-Ub (K6) only, HA- or 6×His-Ub (K11) only, HA- or 6×His-Ub (K27) only, HA- or 6×His-Ub (K33) only, HA- or 6×His-Ub (K48) only, and HA- or 6×His-Ub (K63) only were constructed by cloning the corresponding cDNAs into pcDNA3.1-HA or pCMV-6×His vector by XbaI/NotI or NotI/BamHI sites. pDONR223-KEAP1 constructs (S45F, S102L, E117K, T142M, S144F, R204P, L268P, P278S, P318L, G333C, D389Y, G417R, G419W, R470H, R470S, WW497L, G524C, R601W, G604W, E611D) were purchased from Addgene, and the DNA fragments containing the mutated genes were shuttled into the pCMV-Flag vector by homologous recombination. The pLKO.1-puro lentiviral MISSION shRNA constructs targeting endogenous CUL3 (shCUL3 CDS (TRCN0000307983)): sense, 5'-GACTATATCCAGGGCTTATTG-3', and shCUL3 3'UTR (TRCN0000073343): sense, 5'-CCCTGTTGTAATTTGAGATTT-3'), KEAP1 (shKEAP1 3'UTR (TRCN0000155340)):

sense, 5’-GCACTGCAAATAACCCATCTT-3’, shKEAP1 CDS1 (TRCN0000156676): sense, 5’-GTGGCGAATGATCACAGCAAT-3’, and shKEAP1 CDS2 (TRCN0000155340): sense, 5’-GCGAATGATCACAGCAATGAA-3’), SOX9 (shSOX9 3’UTR (TRCN0000342888)): sense, 5’-GCATCCTTCAATTTCTGTATA-3’, NRF2 (shNRF2 CDS (TRCN0000273494)): sense, 5’-AGTTTGGGAGGAGCTATTATC-3’, and a non-targeting (NT) control shRNA (TRC1/1.5) were from Sigma-Aldrich. For lentiviral expression of SOX9 (WT and mutants), KEAP1 and CKIγ1 in functional studies, corresponding cDNAs were sub-cloned into pSin4-EF2-IRES-Pur lentiviral vector. Lentiviruses were produced by cotransfection of HEK293T cells with recombinant lentivirus vectors and lentivirus packaging plasmids pMD2.G, pRSV-Rev, pMDLGpRRE using a calcium phosphate precipitation-based method. After 48 h of transfection, supernatant containing virus particles was harvested and concentrated. The efficacy of viral each expression of proteins or shRNAs was assessed by western blotting of the target proteins 3 days after infection. Details of plasmid construction are available upon request.

Immunoblotting (IB) and immunoprecipitation (IP)

For the preparartion of Whole-Cell Lysate (WCL), cells were lysed with IP lysis buffer (Cat#87788, Thermo Fisher Scientific) supplemented with protease/phosphatase inhibitors (Cat#5872S, Cell Signaling Technology), incubated on ice for 30 minutes, and cleared by centrifugation at 13,500 rpm at 4 °C for 15 min. The protein concentration were measured by Beckman Coulter DU-800 spectrophotometer using the Bio-Rad protein assay reagent. Lysates were subjected to SDS-PAGE and transferred onto nitrocellulose membranes. The transferred membranes were incubated with primary antibodies at 4°C overnight, followed by

incubation with HRP-conjugated anti-rabbit or anti-mouse or anti-goat secondary antibodies for 2 h at room temperature. Immunoreactive bands were visualized by enhanced chemiluminescence (Cat# 34096, Thermo Fisher Scientific). For immunoprecipitation, cells were collected and lysed in IP lysis buffer supplemented with protease/phosphatase inhibitors, incubated on ice for 30 min, and cleared by centrifugation at 13,500 rpm at 4 °C for 15 min. Total protein lysate (600 μg) was subjected to immunoprecipitation with primary antibodies (2-3 μg) for overnight at 4°C. The primary antibodies were then pulled down with Protein G sepharose beads (Cat# 88848, Thermo Fisher Scientific) by incubating for 3-5 h at 4°C and washing with cold IP lysis buffer for four times. For IB analysis, equal amounts of whole cell lysate (WCL) or immunoprecipitates were separated resolved by 8%-15% SDS-PAGE and immunoblotted probed with indicated antibodies. For immune-reactive bands quantification, signals were densitometrically quantified and normalized to GAPDH or Vinculin with ImageJ software. Relative band intensities values were presented under the corresponding bands in each figures

Real-time RT-PCR analysis

Total RNA was extracted using Trizol reagent (Cat# 9109, TakaRa), and the reverse transcription reaction was performed using RevertAid™ First Strand cDNA Synthesis Kit (Cat# K1622, Fermentas), according to the manufacturer’s instructions. Semi-Quantitative Real-time PCR analysis was performed with SYBR premix Ex Taq (TaKaRa) on an Applied Biosystems 7300 Real Time PCR System supplied with analytical software (Applied Biosystems, USA). Data were normalized to the internal control of GAPDH using the comparative CT method ($2^{-\Delta\Delta CT}$). Averages and standard deviations calculated from at least 3

independent experiments were shown in the figures. Primers used were: SOX9 Forward: 5'-AGCGAACGCACATCAAGAC-3', Reverse: 5'- CTGTAGGCGATCTGTTGGGG -3'; GAPDH Forward: 5'- TGGTATCGTGGAAGGACTC-3', Reverse: 5'-AGTAGAGGCAGGGATGATG-3'.

Colony formation assay

The indicated tumour cells (Huh7 and H1299) were plated in 24-well plates (200 cells per well) and maintained for up to 10-15 days until colonies were visible. Plates were washed with PBS and fixed with 10% acetic aids/10% methanol for 30 min, and then stained with 0.4% crystal violent/20% ethanol. After washing with distilled water, the plates were air-dried and the visible colonies were quantified and analysed as previously described.[2]

Human clinical data analyses

Statistical significance was determined by the log-rank test. For the survival analyses, the median (50th percentile) KEAP1 and SOX9 gene score were used as the threshold. Tumors with a higher gene score than this threshold were categorized as 'high' while the tumors with lower gene score were categorized as 'low'. Raw read counts and normalized counts (sequenced with the Illumina HiSeq platform) for the TCGA (The Cancer Genome Atlas) datasets were downloaded from the Broad Institute GDAC Firehose (https://gdac.broadinstitute.org/) for lung cancer. Information on KEAP1 mutations in the tumors as well as clinical data (including overall survival data for each patient) were downloaded from the cbioportal database (https://www.cbioportal.org/).

In *vivo* ubiquitination assays

HEK293T cells with 80% confluence were co-transfected with HA-, 6×His- or GFP-tagged

ubiquitin and the desired constructs. Thirty-six hours post-transfection, cells were treated with 20 μM MG-132 for 6 h before they were harvested. Cells were lysed in IP lysis buffer containing freshly dissolved iodoacetamide and N-ethylmaleimide (5 mM each) to inhibit deubiquitinating enzymes. Immunoprecipitation was performed using antibodies against the tagged proteins. Immunoprecipitants were washed five times with IP lysis buffer before being resolved by SDS-PAGE and immunoblotted with antibodies against the tags on ubiquitin.

Immunofluorescence (IF) staining

Cells were grown on glass coverslips and fixed with 4% paraformaldehyde for 30 min at room temperature, washed three times with PBS and then permeabilized with 0.05% Triton X-100 for 10 min at room temperature. Following three PBS washes of 5 min each, the coverslips were blocked with 5% BSA for 1 h and then incubated overnight with antibodies against Flag and Myc at 4°C. Following three PBST washes of 10 min each, the coverslips were incubated with secondary antibodies - goat anti-mouse IgG (H+L) conjugated with Alexa Fluor 488 (Life Technologies/Molecular Probes, A11029) and goat anti-rabbit IgG (H+L) conjugated with Alexa Fluor 647 (Life Technologies/Molecular Probes, A32733) for 1 h at room temperature in the dark. Following three PBST washes of 5 min each, the coverslips were stained with 4’, 6’-diamidino-2-phenylindole (DAPI, Sigma-Aldrich, D9542) for 10 min and visualized under a fluorescent microscope.

Reference

[1] C. G. Liu, L. M. Liu, X. J. Chen, J. M. Cheng, H. Zhang, J. J. Shen, J. J. Shan, Y. M. Xu, Z. Yang, M. D. Lai, C. Qian, *Hepatology.* **2016**, *64*, 117.

[2] C. G. Liu, L. M. Liu, X. J. Chen, J. M. Cheng, H. Zhang, C. C. Zhang, J. J. Shan, J. J. Shen, C. Qian, *Cancer Research.* **2018**, *78*, 938.

Table S1. Significantly (adjusted p-value ≤ 0.05) differentially expressed SOX9 target genes used to calculate the SOX9 gene scores in lung cancer.

Entrez ID	Gene Name	$\log_2$ Fold Change	p-value	Expression in Tumors Relative to Normal
23294	ANKS1A	-1.077646049	5.73E-47	Down-Regulated in Tumor
1589	CYP21A2	-1.609816475	1.84E-18	Down-Regulated in Tumor
359845	FAM101B	-1.119586557	2.55E-21	Down-Regulated in Tumor
387680	FAM21A	-0.424448521	3.63E-05	Down-Regulated in Tumor
2210	FCGR1B	-1.031029545	1.42E-10	Down-Regulated in Tumor
474344	GIMAP6	-2.010945736	3.80E-52	Down-Regulated in Tumor
2794	GNL1	-0.18546058	0.000342972	Down-Regulated in Tumor
352961	HCG26	-0.720390306	2.56E-08	Down-Regulated in Tumor
3133	HLA-E	-1.217528911	1.18E-39	Down-Regulated in Tumor
9836	LCMT2	-0.249200087	0.001153631	Down-Regulated in Tumor
3987	LIMS1	-0.19251801	0.019332159	Down-Regulated in Tumor
2122	MECOM	-0.772042487	1.18E-07	Down-Regulated in Tumor
4205	MEF2A	-0.817488973	2.00E-48	Down-Regulated in Tumor
25832	NBPF14	-0.40000942	0.001206512	Down-Regulated in Tumor
197135	PATL2	-0.516373424	0.001362036	Down-Regulated in Tumor
22937	SCAP	-0.160817391	0.009327222	Down-Regulated in Tumor
6457	SH3GL3	-5.028376286	3.28E-86	Down-Regulated in Tumor
115286	SLC25A26	-0.157028076	0.006871538	Down-Regulated in Tumor
7124	TNF	-0.700844424	0.000829206	Down-Regulated in Tumor
7148	TNXB	-3.132237092	2.26E-57	Down-Regulated in Tumor
286753	TUSC5	-0.886508945	0.024147028	Down-Regulated in Tumor
10163	WASF2	-0.513648766	2.79E-12	Down-Regulated in Tumor
1182	CLCN3	0.253729674	0.000471287	Up-Regulated in Tumor
9075	CLDN2	1.38292664	7.40E-06	Up-Regulated in Tumor
1306	COL15A1	1.309732696	1.49E-11	Up-Regulated in Tumor
1734	DIO2	2.652813997	8.85E-36	Up-Regulated in Tumor
10919	EHMT2	0.559918122	3.21E-13	Up-Regulated in Tumor
8893	EIF2B5	0.215668472	2.22E-06	Up-Regulated in Tumor
83715	ESPN	2.130420315	1.05E-18	Up-Regulated in Tumor
158584	FAAH2	0.702165803	1.24E-08	Up-Regulated in Tumor
22868	FASTKD2	0.230008756	1.75E-05	Up-Regulated in Tumor
51463	GPR89B	0.608521625	0.000412527	Up-Regulated in Tumor
3670	ISL1	3.49469619	9.57E-14	Up-Regulated in Tumor
3973	LHCGR	2.328800103	9.77E-07	Up-Regulated in Tumor
57819	LSM2	0.689891562	7.00E-15	Up-Regulated in Tumor

79157	MFSD11	0.15342387	0.021838301	Up-Regulated in Tumor
145282	MIPOL1	0.824957629	1.73E-18	Up-Regulated in Tumor
4477	MSMB	1.538649149	0.00042008	Up-Regulated in Tumor
5704	PSMC4	0.771352761	7.23E-27	Up-Regulated in Tumor
80352	RNF39	1.157639175	5.43E-09	Up-Regulated in Tumor
339665	SLC35E4	0.892744013	1.99E-14	Up-Regulated in Tumor
724102	SNHG4	1.321798233	2.34E-11	Up-Regulated in Tumor
6941	TCF19	1.010871554	7.55E-19	Up-Regulated in Tumor
7268	TTC4	0.394959393	5.75E-13	Up-Regulated in Tumor
7407	VARS	1.179465796	5.47E-33	Up-Regulated in Tumor
81030	ZBP1	0.967810779	5.82E-09	Up-Regulated in Tumor
51538	ZCCHC17	0.232971761	0.000369073	Up-Regulated in Tumor
346171	ZFP57	0.754750857	0.007182967	Up-Regulated in Tumor

Table S2. Gene sets used in the KEAP1 mutant gene score calculation.

Up-regulated gene (Entrez ID)	213, 481, 796, 873, 1048, 1114, 1277, 1278, 1281, 1363, 1373, 1645, 1646, 1728, 1832, 1937, 2243, 2244, 2266, 2597, 2923, 2950, 3149, 3481, 3488, 3875, 4477, 4736, 4922, 4953, 5045, 5122, 5126, 5479, 6122, 6124, 6125, 6130, 6132, 6136, 6137, 6141, 6154, 6155, 6156, 6157, 6160, 6167, 6168, 6171, 6175, 6176, 6188, 6193, 6194, 6201, 6202, 6203, 6204, 6205, 6208, 6210, 6217, 6218, 6222, 6223, 6224, 6227, 6229, 6280, 6286, 6337, 6542, 6590, 7033, 7178, 7184, 7296, 7494, 8644, 10232, 10551, 23521, 26986, 51297, 56892, 96610, 125144, 283120, 388524, 727897
Down-regulated gene (Entrez ID)	2, 60, 301, 302, 334, 348, 351, 358, 397, 567, 677, 710, 712, 713, 714, 718, 720, 754, 824, 960, 966, 968, 972, 975, 999, 1075, 1116, 1364, 1368, 1508, 1509, 1510, 1512, 1520, 1522, 1634, 1718, 1755, 2023, 2316, 2335, 2495, 2512, 2752, 2771, 2896, 2934, 3105, 3106, 3107, 3108, 3109, 3113, 3115, 3117, 3119, 3122, 3123, 3127, 3133, 3315, 3339, 3371, 3383, 3423, 3429, 3512, 3675, 3689, 3880, 3936, 3958, 3959, 4035, 4061, 4069, 4070, 4233, 4478, 4582, 4627, 4680, 5265, 5284, 5358, 5660, 5792, 6035, 6382, 6385, 6414, 6440, 6441, 6696, 6772, 6892, 7045, 7052, 7078, 7117, 7145, 7316, 7356, 7430, 7431, 7453, 7503, 7805, 8407, 8878, 9445, 9476, 9961, 10406, 10437, 10457, 10568, 10577, 10628, 23524, 23654, 30061, 56241, 57674, 79026, 79098, 79888, 92304, 146556, 221395, 283131, 375790

Table S3. A list of the SOX9 and KEAP1-mutant gene scores with the clinical and mutation data.

Patient.ID	KEAP1.up.score	KEAP1.down.score	KEAP1.final.score	KEAP1.mutant	protein.change	SOX9.up.score	SOX9.down.score	SOX9.final.score	Stage	Survival Time
1592	2.758383	2.337535	-0.42085	Non-Mutant	NA	0.457823	0.706055	-0.24823	Stage IA	23.04632
1594	2.480097	2.482728	0.002631	Mutant	R470H	0.519981	0.410866	0.109115	Stage IIIA	38.72834
1595	2.67902	2.23646	-0.44256	Non-Mutant	NA	0.493543	0.721629	-0.22809	Stage IA	48.62412
1596	2.393616	2.41635	0.022733	Non-Mutant	NA	0.709546	0.452185	0.257361	Stage IIB	67.88967
1676	2.711977	2.072881	-0.6391	Non-Mutant	NA	0.709546	0.452185	0.257361	Stage IA	56.81034
1677	2.62462	2.306054	-0.31857	Non-Mutant	NA	0.484465	0.387435	0.09703	Stage IIIA	20.64635
1678	2.028983	2.549343	0.52036	Non-Mutant	NA	0.609234	0.031503	0.577732	Stage IIA	39.08998
1679	2.699043	2.234064	-0.46498	Non-Mutant	NA	0.563429	0.655185	-0.09176	Stage IIIA	81.79636
1680	2.753027	2.307705	-0.44532	Non-Mutant	NA	0.520187	0.43895	0.081237	Stage IV	37.01877
1681	2.844339	2.294655	-0.54968	Non-Mutant	NA	0.472343	0.638513	-0.16617	Stage IA	38.3667
2655	2.787874	2.395676	-0.3922	Mutant	S144F	0.542336	0.684513	-0.14218	Stage IA	43.52829
2656	2.81058	2.204516	-0.60606	Non-Mutant	NA	0.194423	0.690808	-0.49639	Stage IB	46.98031
2657	2.884323	2.257022	-0.6273	Non-Mutant	NA	0.532016	0.721037	-0.18902	Stage IB	44.41595
2659	2.754076	2.134138	-0.61994	Non-Mutant	NA	0.420492	0.896613	-0.47612	Stage IIB	44.94197
2662	2.604098	2.11736	-0.48674	Non-Mutant	NA	0.448525	0.454979	-0.00645	Stage IB	42.08173
2665	2.676861	2.199811	-0.47705	Non-Mutant	NA	0.525884	0.476001	0.049883	Stage IIB	42.77213
2666	2.695259	2.314611	-0.38065	Non-Mutant	NA	0.304618	0.722242	-0.41762	Stage IB	3.189006
2668	2.615881	2.004999	-0.61088	Non-Mutant	NA	0.505225	0.713644	-0.20842	Stage IB	25.0189
3396	2.702535	2.157358	-0.54518	Non-Mutant	NA	0.450791	0.653746	-0.20296	Stage IIIA	37.15028
3398	2.791491	2.281873	-0.50962	Non-Mutant	NA	0.461395	0.540225	-0.07883	Stage IA	38.2352
3615	2.646214	2.444773	-0.20144	Mutant	E449*	0.526896	0.593061	-0.06617	Stage IB	0.460269
3770	2.816337	2.236102	-0.58023	Non-Mutant	NA	0.328916	0.460662	-0.13175	Stage IA	20.05457
3771	2.599942	2.428378	-0.17156	Mutant	G417E	0.371972	0.548795	-0.17682	Stage IA	20.05457
3772	2.815098	2.254001	-0.5611	Non-Mutant	NA	0.675245	0.42328	0.251965	Stage IB	18.83815

3773	2.80115	2.177112	-0.62404	Non-Mutant	NA	0.240809	0.657873	-0.41706	Stage IB	14.0382
3774	2.816367	2.433269	-0.3831	Non-Mutant	NA	0.434932	0.77135	-0.33642	Stage IB	12.6574
3918	2.740711	2.265992	-0.47472	Non-Mutant	NA	0.456922	0.656009	-0.19909	Stage IA	34.0599
3919	2.848322	2.284872	-0.56345	Non-Mutant	NA	0.413055	0.729914	-0.31686	Stage IA	33.73114
4112	2.639359	2.195578	-0.44378	Non-Mutant	NA	0.474948	0.425381	0.049567	Stage IB	26.56409
4122	2.643669	2.146449	-0.49722	Non-Mutant	NA	0.51471	0.487817	0.026893	Stage IA	7.397179
4123	2.556518	2.064717	-0.4918	Non-Mutant	NA	0.468083	0.376659	0.091425	Stage IA	5.983496
4244	2.783124	2.113804	-0.66932	Non-Mutant	NA	0.292213	0.498919	-0.20671	Stage IV	0
4249	2.776531	2.103494	-0.67304	Non-Mutant	NA	0.463545	0.742934	-0.27939	Stage IB	50.07068
4250	2.692826	2.113754	-0.57907	Non-Mutant	NA	0.460646	0.493836	-0.03319	Stage IIIA	3.978039
4382	2.659674	2.322944	-0.33673	Non-Mutant	NA	0.376427	0.628717	-0.25229	Stage IB	19.95595
4384	2.728598	2.548624	-0.17997	Mutant	G524C	0.394904	0.760944	-0.36604	Stage IIIA	14.00533
4389	2.668762	2.446205	-0.22256	Mutant	F246L	0.610293	0.539751	0.070543	Stage IA	45.00773
4390	2.45293	2.6767	0.22377	Non-Mutant	NA	0.775037	0.350792	0.424245	Stage IB	37.01877
4395	2.412175	2.469045	0.05687	Mutant	D479G	0.459378	0.427603	0.031774	Stage IIIB	0
4396	2.562276	2.599322	0.037046	Non-Mutant	NA	0.63594	0.734473	-0.09853	Stage IIIB	9.961535
4397	2.444214	2.108538	-0.33568	Non-Mutant	NA	0.511153	0.488126	0.023026	Stage IIB	24.03261
4398	2.684661	2.096179	-0.58848	Non-Mutant	NA	0.472522	0.537731	-0.06521	Stage IIIB	47.04606
4402	2.811148	2.29659	-0.51456	Non-Mutant	NA	0.577926	0.541087	0.036839	Stage IV	8.02183
4403	2.768489	2.363698	-0.40479	Non-Mutant	NA	0.416143	0.715961	-0.29982	Stage IB	19.00253
4405	2.809304	2.183224	-0.62608	Non-Mutant	NA	0.429165	0.689916	-0.26075	Stage IB	20.05457
4410	2.802946	2.165324	-0.63762	Non-Mutant	NA	0.490115	0.704206	-0.21409	Stage IB	0
4415	2.222772	2.413487	0.190715	Non-Mutant	NA	0.272576	0.447619	-0.17504	Stage IIIB	2.991748
4417	2.716946	2.618923	-0.09802	Non-Mutant	NA	0.571942	0.591902	-0.01996	Stage IB	14.95874
4418	2.523691	2.591471	0.06778	Mutant	R460G	0.471852	0.428904	0.042947	Stage IIIA	9.00812
4420	2.341575	2.479107	0.137532	Non-Mutant	NA	0.653207	0.450774	0.202433	Stage IB	29.98323
4422	2.629166	2.436787	-0.19238	Non-Mutant	NA	0.383007	0.637724	-0.25472	Stage IB	11.99987

4424	2.764851	2.304966	-0.45988	Non-Mutant	NA	0.398751	0.777142	-0.37839	Stage IIB	30.01611
4425	2.796818	2.269475	-0.52734	Non-Mutant	NA	0.578013	0.543967	0.034046	Stage IV	21.99428
4426	2.702363	2.076813	-0.62555	Non-Mutant	NA	0.341286	0.592491	-0.25121	Stage IB	26.00519
4427	2.688005	2.011453	-0.67655	Non-Mutant	NA	0.526924	0.759037	-0.23211	Stage IIB	26.00519
4430	2.838695	2.390662	-0.44803	Non-Mutant	NA	0.44134	0.756194	-0.31485	Stage IB	25.0189
4432	2.658375	2.451503	-0.20687	Non-Mutant	NA	0.608545	0.473836	0.134709	Stage IIB	25.0189
4433	2.762217	2.294764	-0.46745	Non-Mutant	NA	0.511074	0.874705	-0.36363	Stage IB	23.99974
4434	2.837018	2.364386	-0.47263	Non-Mutant	NA	0.504274	0.721604	-0.21733	Stage IV	15.02449
4486	2.540663	2.406067	-0.1346	Mutant	G333S	0.416814	0.387832	0.028982	Stage IA	76.20738
4487	2.706293	2.439697	-0.2666	Non-Mutant	NA	0.47315	0.594124	-0.12097	Stage IA	28.10928
4488	2.678174	2.256454	-0.42172	Mutant	Q284L	0.453449	0.501904	-0.04845	Stage IA	28.56955
4490	2.742654	2.265316	-0.47734	Non-Mutant	NA	0.513518	0.561638	-0.04812	Stage IIIA	12.6574
4494	2.728838	2.45104	-0.2778	Non-Mutant	NA	0.447741	0.36558	0.082161	Stage IIIA	35.53934
4501	2.818391	2.224148	-0.59424	Non-Mutant	NA	0.667117	0.580179	0.086938	Stage IB	46.7173
4505	2.816307	2.209077	-0.60723	Non-Mutant	NA	0.464956	0.65373	-0.18877	Stage IIB	14.07108
4506	2.388383	2.512628	0.124245	Mutant	M503K	0.525451	0.476693	0.048758	Stage IIB	32.84348
4507	2.516796	2.516557	-0.00024	Mutant	R470C	0.464973	0.480611	-0.01564	Stage IIIA	8.810862
4510	2.709371	2.555147	-0.15422	Non-Mutant	NA	0.459349	0.684457	-0.22511	Stage IIB	29.45721
4512	2.774316	2.151419	-0.6229	Non-Mutant	NA	0.493023	0.508884	-0.01586	Stage IIIA	29.7531
4514	2.611829	2.613986	0.002157	Mutant	R470S	0.901207	0.289767	0.61144	Stage IA	55.8898
4625	2.643657	2.047413	-0.59624	Non-Mutant	NA	0.659105	0.523356	0.13575	Stage IB	97.74139
4626	2.85636	2.238628	-0.61773	Non-Mutant	NA	0.199319	0.873043	-0.67372	Stage IIA	120.7877
4627	2.721556	2.34817	-0.37339	Non-Mutant	NA	0.481295	0.793904	-0.31261	Stage IIA	37.70918
4628	2.643137	2.140313	-0.50282	Non-Mutant	NA	0.518067	0.263667	0.2544	Stage IIB	49.05152
4631	2.252246	2.442708	0.190462	Mutant	F139L	0.519157	0.333534	0.185623	Stage IB	11.63823
4632	2.693033	2.272599	-0.42043	Non-Mutant	NA	0.509251	0.380615	0.128636	Stage IV	44.61321
4658	2.805205	2.219979	-0.58523	Non-Mutant	NA	0.436778	0.650005	-0.21323	Stage IB	52.60216

4659	2.740022	2.666009	-0.07401	Non-Mutant	NA	0.51179	0.435258	0.076533	Stage IIIA	23.37509
4662	2.751755	2.104576	-0.64718	Non-Mutant	NA	0.410654	0.577027	-0.16637	Stage IA	82.68403
4666	2.68697	2.192326	-0.49464	Non-Mutant	NA	0.532169	0.615474	-0.08331	Stage IV	26.30108
4668	2.627982	2.336363	-0.29162	Non-Mutant	NA	0.617407	0.535967	0.08144	Stage IIB	15.35326
4670	2.437731	2.618444	0.180713	Non-Mutant	NA	0.625414	0.450243	0.175171	Stage IV	4.306802
4675	2.636385	2.413652	-0.22273	Non-Mutant	NA	0.388875	0.483236	-0.09436	Stage IIIA	30.312
4676	2.675078	2.275983	-0.3991	Mutant	R415C	0.379428	0.339962	0.039465	Stage IIA	9.238255
4677	2.729801	2.502524	-0.22728	Non-Mutant	NA	0.358091	0.667263	-0.30917	Stage IA	1.249301
5044	2.723079	2.195347	-0.52773	Non-Mutant	NA	0.426631	0.454857	-0.02823	Stage IIIB	20.51484
5045	2.827334	2.265865	-0.56147	Non-Mutant	NA	0.378227	0.547215	-0.16899	Stage IA	71.47319
5049	2.772114	2.206281	-0.56583	Non-Mutant	NA	0.470632	0.730972	-0.26034	Stage IA	101.7194
5051	2.551722	2.612866	0.061143	Mutant	G417R	0.425887	0.410648	0.015239	Stage IIIA	15.7149
5055	2.81521	2.237056	-0.57815	Non-Mutant	NA	0.456047	0.687009	-0.23096	Stage IIA	60.16372
5066	2.579338	2.272351	-0.30699	Non-Mutant	NA	0.420853	0.492383	-0.07153	Stage IB	47.4077
5068	2.731696	2.178119	-0.55358	Non-Mutant	NA	0.41543	0.355335	0.060095	Stage IIB	49.28165
5072	2.63924	2.663387	0.024146	Non-Mutant	NA	0.513557	0.534773	-0.02122	Stage IIIA	8.219088
5122	2.660266	2.123004	-0.53726	Non-Mutant	NA	0.371726	0.846232	-0.47451	Stage IB	NA
5125	2.713549	1.987314	-0.72623	Non-Mutant	NA	0.435692	0.623207	-0.18752	Stage IIB	66.64037
5126	2.830254	2.27331	-0.55694	Non-Mutant	NA	0.472108	0.638053	-0.16595	Stage IIIA	NA
5146	2.708751	2.32111	-0.38764	Non-Mutant	NA	0.584297	0.571613	0.012684	Stage IB	77.8512
5147	2.697067	2.391015	-0.30605	Non-Mutant	NA	0.619208	0.473655	0.145553	Stage IB	43.82418
5375	2.372109	2.348205	-0.0239	Non-Mutant	NA	0.476304	0.265028	0.211275	Stage IIIA	8.679357
5423	2.783401	2.201161	-0.58224	Non-Mutant	NA	0.245264	0.407823	-0.16256	Stage IIB	4.964329
5425	2.727415	2.464537	-0.26288	Non-Mutant	NA	0.681896	0.674197	0.007699	Stage IIB	28.99694
5428	2.498603	2.27595	-0.22265	Non-Mutant	NA	0.442979	0.431939	0.01104	Stage IIA	22.02716
5429	2.384896	2.311014	-0.07388	Non-Mutant	NA	0.808347	0.279005	0.529341	Stage IIIA	9.040997
5607	2.793633	2.242536	-0.5511	Non-Mutant	NA	0.483627	0.700975	-0.21735	Stage IIB	NA

5608	2.657007	2.680441	0.023434	Non-Mutant	NA	0.665235	0.446919	0.218317	Stage IA	93.10583
5611	2.529817	2.23327	-0.29655	Non-Mutant	NA	0.388277	0.429622	-0.04135	Stage IB	85.31413
5643	2.367584	2.247347	-0.12024	Non-Mutant	NA	0.426637	0.572366	-0.14573	Stage IIIA	33.30374
5644	2.170634	2.575428	0.404794	Non-Mutant	NA	0.380496	0.229484	0.151012	Stage IB	28.37229
5645	2.838582	2.112657	-0.72593	Non-Mutant	NA	0.299102	0.847277	-0.54817	Stage IA	28.01065
5715	2.721177	2.350103	-0.37107	Non-Mutant	NA	0.592111	0.380011	0.2121	Stage IB	2.038334
5774	2.226147	2.544767	0.318619	Non-Mutant	NA	0.574519	0.321639	0.25288	Stage IB	87.97712
5775	2.187564	1.93414	-0.25342	Non-Mutant	NA	0.361244	0.707219	-0.34597	Stage IIIA	2.038334
5778	2.785094	2.168388	-0.61671	Non-Mutant	NA	0.413504	0.462717	-0.04921	Stage IB	42.90364
5779	2.331212	2.244217	-0.08699	Non-Mutant	NA	0.468377	0.59314	-0.12476	Stage IIIA	28.40517
5781	2.485625	2.517345	0.03172	Mutant	V99L	0.661173	0.393031	0.268142	Stage IB	51.25423
5815	2.746853	2.231122	-0.51573	Non-Mutant	NA	0.418551	0.578875	-0.16032	Stage IIB	28.47092
5899	2.365166	2.397076	0.031909	Non-Mutant	NA	0.539401	0.324362	0.215039	Stage IA	30.57501
5908	2.614482	2.108917	-0.50557	Non-Mutant	NA	0.568138	0.547873	0.020265	Stage IA	27.09011
5930	2.686826	2.609663	-0.07716	Non-Mutant	NA	0.604126	0.616989	-0.01286	Stage IIIA	9.271131
5931	2.139817	2.168277	0.028461	Non-Mutant	NA	0.490063	0.535019	-0.04496	Stage IB	14.26834
5932	2.614925	2.330873	-0.28405	Non-Mutant	NA	0.510405	0.641229	-0.13082	Stage IIB	40.60229
5933	2.62464	1.989088	-0.63555	Non-Mutant	NA	0.389472	0.613006	-0.22353	Stage IIIB	78.67311
5935	2.782814	2.387386	-0.39543	Non-Mutant	NA	0.35887	0.692925	-0.33406	Stage IA	21.46826
5936	2.628237	2.562559	-0.06568	Mutant	Q46*	0.459471	0.579697	-0.12023	Stage IIIA	8.449222
5939	2.718566	2.451813	-0.26675	Mutant	R261P	0.410879	0.792551	-0.38167	Stage IB	15.12312
5941	2.850708	2.235024	-0.61568	Non-Mutant	NA	0.697745	0.771788	-0.07404	Stage IIIA	48.45974
5942	2.771212	2.265448	-0.50576	Non-Mutant	NA	0.377233	0.817619	-0.44039	Stage IA	60.72262
5944	2.833583	2.116568	-0.71702	Non-Mutant	NA	0.536222	0.798729	-0.26251	Stage IA	57.53362
5946	2.407706	2.234907	-0.1728	Non-Mutant	NA	0.426599	0.774165	-0.34757	Stage IA	53.16106
6145	2.775047	2.489098	-0.28595	Non-Mutant	NA	0.66774	0.71071	-0.04297	Stage IA	19.56143
6146	2.493264	2.261023	-0.23224	Non-Mutant	NA	0.466277	0.660056	-0.19378	Stage IIB	23.93398

6148	2.772912	2.249356	-0.52356	Non-Mutant	NA	0.540913	0.714385	-0.17347	Stage IA	23.14495
6178	2.787151	2.22237	-0.56478	Non-Mutant	NA	0.568763	0.591316	-0.02255	Stage IIIA	14.72861
6203	2.863319	2.252247	-0.61107	Non-Mutant	NA	0.190468	1.031503	-0.84103	Stage IIIA	NA
6205	2.726379	2.159805	-0.56657	Non-Mutant	NA	0.500605	0.737918	-0.23731	Stage IB	NA
6206	2.679048	2.378095	-0.30095	Non-Mutant	NA	0.375496	0.746424	-0.37093	Stage IB	85.14975
6207	2.646835	2.180921	-0.46591	Non-Mutant	NA	0.380039	0.472344	-0.0923	Stage IIIA	NA
6211	2.413442	2.54037	0.126928	Mutant	R204P	0.479832	0.321728	0.158104	Stage IB	NA
6212	2.84133	2.261803	-0.57953	Non-Mutant	NA	0.459382	0.614234	-0.15485	Stage IIB	49.84055
6214	2.47502	2.298445	-0.17657	Non-Mutant	NA	0.685237	0.664263	0.020973	Stage IIIA	36.65713
6215	2.749393	2.20444	-0.54495	Non-Mutant	NA	0.314298	0.532153	-0.21785	Stage IB	5.720485
6216	2.806441	2.14916	-0.65728	Non-Mutant	NA	0.40064	0.565517	-0.16488	Stage IA	4.635566
6217	2.836427	2.129059	-0.70737	Non-Mutant	NA	0.348847	0.685801	-0.33695	Stage IIA	13.87382
6543	2.816445	2.279445	-0.537	Non-Mutant	NA	0.230141	0.590045	-0.3599	Stage IA	14.30121
6562	2.722149	2.265858	-0.45629	Non-Mutant	NA	0.351168	0.585126	-0.23396	Stage IIA	12.36151
6590	2.504104	2.088289	-0.41581	Non-Mutant	NA	0.471962	0.79177	-0.31981	Stage IB	42.34474
6591	1.924861	2.046	0.121139	Non-Mutant	NA	0.435126	0.560528	-0.1254	Stage IV	3.912286
6592	2.577925	2.427593	-0.15033	Non-Mutant	NA	0.574723	0.589494	-0.01477	Stage IB	25.54493
6593	2.844322	2.316596	-0.52773	Non-Mutant	NA	0.615239	0.645739	-0.0305	Stage IIIA	11.04645
6594	2.543778	1.941489	-0.60229	Non-Mutant	NA	0.44318	0.21154	0.23164	Stage IIIA	12.16425
6595	2.608863	2.199371	-0.40949	Non-Mutant	NA	0.566013	0.557487	0.008526	Stage IIIA	6.213631
6597	2.661371	2.161342	-0.50003	Non-Mutant	NA	0.422559	0.360454	0.062105	Stage IB	41.68721
6642	2.599771	2.517961	-0.08181	Non-Mutant	NA	0.571208	0.721332	-0.15012	Stage IB	80.51419
6673	2.761977	2.334044	-0.42793	Non-Mutant	NA	0.47857	0.687922	-0.20935	Stage I	0.72328
6712	2.741655	2.170265	-0.57139	Non-Mutant	NA	0.462014	0.581293	-0.11928	Stage IIA	5.621856
6725	2.646828	2.112612	-0.53422	Non-Mutant	NA	0.33316	0.636916	-0.30376	Stage IB	8.416346
6742	2.625205	2.603253	-0.02195	Mutant	L268P	0.648688	0.557509	0.091178	Stage IIA	16.04366
6743	2.651016	2.434303	-0.21671	Non-Mutant	NA	0.680442	0.50346	0.176982	Stage IIIA	53.29257

6744	2.85373	2.291355	-0.56238	Non-Mutant	NA	0.418713	0.72674	-0.30803	Stage IIA	55.3309
6745	2.791303	2.127365	-0.66394	Non-Mutant	NA	0.38849	0.703186	-0.3147	Stage IIIA	17.16146
6761	2.653794	2.07809	-0.5757	Non-Mutant	NA	0.488674	0.386297	0.102378	Stage IIIA	11.63823
6767	2.484543	2.025187	-0.45936	Non-Mutant	NA	0.565952	0.570812	-0.00486	Stage IIB	22.25729
6774	2.622047	2.321547	-0.3005	Non-Mutant	NA	0.509092	0.787898	-0.27881	Stage IIIA	21.63264
6775	2.789833	2.152528	-0.6373	Non-Mutant	NA	0.498799	0.595939	-0.09714	Stage IB	23.17783
6776	2.649829	2.406723	-0.24311	Mutant	V155A	0.505767	0.630272	-0.1245	Stage IA	86.00454
6777	2.80566	2.220788	-0.58487	Non-Mutant	NA	0.358298	0.831526	-0.47323	Stage IB	32.44896
6778	2.763451	2.369118	-0.39433	Non-Mutant	NA	0.19209	0.89071	-0.69862	Stage IA	61.28152
6779	2.596385	2.284499	-0.31189	Mutant	T142M	0.640512	0.73526	-0.09475	Stage IIB	16.43818
6828	2.716643	2.453448	-0.2632	Non-Mutant	NA	0.42739	0.82411	-0.39672	Stage IA	10.61906
6829	2.5797	2.098696	-0.481	Non-Mutant	NA	0.59818	0.78251	-0.18433	Stage IB	41.35845
6830	2.777244	2.416288	-0.36096	Non-Mutant	NA	0.36973	0.722213	-0.35248	Stage IIA	1.972581
6831	2.435199	2.414071	-0.02113	Non-Mutant	NA	0.607273	0.524491	0.082781	Stage IB	10.19167
6835	2.808691	2.133578	-0.67511	Non-Mutant	NA	0.280042	0.83419	-0.55415	Stage IA	2.597232
6836	2.369172	1.954401	-0.41477	Non-Mutant	NA	0.350391	0.267055	0.083336	Stage IB	13.70944
6840	2.637749	2.261866	-0.37588	Non-Mutant	NA	0.442582	0.781859	-0.33928	Stage IA	12.23
6847	1.90638	2.068073	0.161693	Non-Mutant	NA	0.564988	0.536383	0.028606	Stage IB	27.68189
6848	2.393779	1.976804	-0.41697	Non-Mutant	NA	0.483145	0.908001	-0.42486	Stage IIIA	7.364303
6849	2.767774	2.412171	-0.3556	Mutant	A159P	0.465081	0.62953	-0.16445	Stage IIIA	1.150672
6851	2.763662	2.120118	-0.64354	Non-Mutant	NA	0.48115	0.817346	-0.3362	Stage IIA	5.884867
6968	2.44276	2.283458	-0.1593	Non-Mutant	NA	0.427908	0.403778	0.024131	Stage IV	42.50912
6969	2.552634	2.272436	-0.2802	Non-Mutant	NA	0.583388	0.521451	0.061937	Stage IB	40.7338
6970	2.739052	2.570342	-0.16871	Mutant	K323*	0.57668	0.735818	-0.15914	Stage IIIA	15.25463
6971	2.809157	2.526627	-0.28253	Non-Mutant	NA	0.610571	0.758507	-0.14794	Stage IB	46.02689
6972	2.261597	2.330632	0.069035	Mutant	X570_splice	0.500732	0.488785	0.011947	Stage IB	53.65421
6975	2.344025	2.546652	0.202628	Non-Mutant	NA	0.49783	0.574324	-0.07649	Stage IIB	3.87941

6978	2.635631	1.997646	-0.63798	Non-Mutant	NA	0.3287	0.661211	-0.33251	Stage IIA	5.786238
6979	2.80742	2.193178	-0.61424	Non-Mutant	NA	0.474909	0.712002	-0.23709	Stage IIB	7.791695
6980	2.784139	2.189305	-0.59483	Non-Mutant	NA	0.554216	0.70405	-0.14983	Stage IA	69.33623
6981	2.739258	2.130453	-0.60881	Non-Mutant	NA	0.435064	0.365492	0.069572	Stage IIIA	45.33649
6982	2.810858	2.239245	-0.57161	Non-Mutant	NA	0.535134	0.353503	0.181631	Stage IIB	32.71197
6983	2.747465	2.667234	-0.08023	Non-Mutant	NA	0.682717	0.526641	0.156076	Stage IIB	92.80994
6984	2.565077	2.334508	-0.23057	Non-Mutant	NA	0.553343	0.54137	0.011973	Stage IIB	24.98603
6985	2.847624	2.36593	-0.48169	Non-Mutant	NA	0.63242	0.597786	0.034634	Stage IB	40.53654
6986	2.764322	2.217565	-0.54676	Non-Mutant	NA	0.30493	0.699545	-0.39462	Stage IB	107.2098
6987	2.792482	2.460461	-0.33202	Non-Mutant	NA	0.420912	0.743711	-0.3228	Stage IA	70.25676
7025	2.82256	2.264603	-0.55796	Non-Mutant	NA	0.5003	0.713614	-0.21331	Stage IB	108.6563
7027	2.415268	2.545052	0.129784	Mutant	R320W	0.428452	0.472537	-0.04408	Stage IB	100.5688
7030	2.79857	2.379996	-0.41857	Non-Mutant	NA	0.403355	0.760919	-0.35756	Stage IIB	NA
7031	2.703407	2.485829	-0.21758	Non-Mutant	NA	0.541217	0.566501	-0.02528	Stage IB	NA
7039	2.74745	2.214451	-0.533	Non-Mutant	NA	0.448156	0.640411	-0.19225	Stage IIB	41.81872
7043	2.35862	2.32519	-0.03343	Mutant	L100P	0.49147	0.470899	0.020571	Stage IA	16.53681
7109	2.570648	2.387366	-0.18328	Non-Mutant	NA	0.500402	0.669971	-0.16957	Stage IA	10.12592
7143	2.796551	2.075514	-0.72104	Non-Mutant	NA	0.298534	0.487523	-0.18899	Stage IB	163.0996
7145	2.699112	2.131966	-0.56715	Non-Mutant	NA	0.503401	0.525177	-0.02178	Stage IV	27.15587
7146	2.480089	2.143983	-0.33611	Non-Mutant	NA	0.363648	0.543208	-0.17956	Stage IIIA	5.687609
7147	2.791443	2.211473	-0.57997	Non-Mutant	NA	0.58215	0.516567	0.065583	Stage IIB	19.26554
7148	2.681172	2.704032	0.02286	Non-Mutant	NA	0.580403	0.488006	0.092398	Stage IIB	20.5806
7149	2.560319	2.564535	0.004216	Mutant	W497L	0.425058	0.645594	-0.22054	Stage IIIB	129.5328
7150	2.308543	2.564024	0.255481	Non-Mutant	NA	0.575332	0.388162	0.18717	Stage IIB	21.89565
7152	2.736848	2.432254	-0.30459	Non-Mutant	NA	0.436582	0.69236	-0.25578	Stage IB	39.94477
7153	2.561706	2.532481	-0.02922	Mutant	E117K	0.620024	0.455733	0.164292	Stage IB	119.5055
7154	2.510497	2.347202	-0.16329	Mutant	W252C	0.309543	0.400596	-0.09105	Stage IIIA	19.49568

7155	1.905659	1.971649	0.06599	Non-Mutant	NA	0.485864	0.63005	-0.14419	Stage IB	38.49821
7156	2.418724	2.409192	-0.00953	Mutant	G477Afs*23	0.213935	0.646523	-0.43259	Stage IV	32.08732
7158	2.643362	2.133953	-0.50941	Mutant	I461V	0.522758	0.627417	-0.10466	Stage IIIB	5.884867
7159	2.449675	2.596093	0.146418	Non-Mutant	NA	0.669474	0.596273	0.0732	Stage IA	64.89792
7160	2.756886	2.610877	-0.14601	Non-Mutant	NA	0.498393	0.744843	-0.24645	Stage IV	22.91482
7161	2.383439	2.599852	0.216413	Non-Mutant	NA	0.658589	0.59462	0.063969	Stage IIB	9.567018
7162	2.756974	2.467339	-0.28963	Non-Mutant	NA	0.442479	0.886886	-0.44441	Stage IA	104.1852
7163	2.481996	2.192064	-0.28993	Non-Mutant	NA	0.361117	0.410156	-0.04904	Stage IB	238.2878
7166	2.374213	2.65145	0.277237	Mutant	F280Y	0.444566	0.311337	0.133229	Stage IIB	8.482099
7167	2.512386	2.431705	-0.08068	Mutant	G480W	0.446455	0.482341	-0.03589	Stage IV	88.1415
7220	2.374926	2.62548	0.250554	Non-Mutant	NA	0.473408	0.605437	-0.13203	Stage IIIA	26.53122
7227	2.795011	2.194958	-0.60005	Non-Mutant	NA	0.357191	0.760949	-0.40376	Stage IIIA	31.29829
7271	2.842387	2.321173	-0.52121	Non-Mutant	NA	0.311434	0.82736	-0.51593	Stage IA	26.30108
7281	2.815231	2.137368	-0.67786	Non-Mutant	NA	0.427895	0.45703	-0.02914	Stage IA	28.66818
7283	2.796644	2.247991	-0.54865	Non-Mutant	NA	0.460083	0.868175	-0.40809	Stage IIIA	20.0217
7284	2.768301	2.272882	-0.49542	Non-Mutant	NA	0.468882	0.865089	-0.39621	Stage IIB	7.988954
7347	2.872589	2.320235	-0.55235	Non-Mutant	NA	0.404823	0.7626	-0.35778	Stage IA	22.45455
7348	2.800296	2.312036	-0.48826	Non-Mutant	NA	0.524331	0.491961	0.03237	Stage IA	17.45734
7458	2.871355	2.214068	-0.65729	Non-Mutant	NA	0.339721	0.832079	-0.49236	Stage IIIA	24.55863
7498	2.726467	2.466018	-0.26045	Mutant	E218V	0.344741	0.819487	-0.47475	Stage IA	39.08998
7499	2.744627	2.250158	-0.49447	Non-Mutant	NA	0.69937	0.381865	0.317504	Stage IB	50.33369
7535	2.690993	2.213737	-0.47726	Non-Mutant	NA	0.359593	0.387315	-0.02772	Stage IB	31.19966
7536	2.350174	2.436628	0.086454	Non-Mutant	NA	0.557041	0.464292	0.092748	Stage IIIA	8.02183
7537	2.681191	2.324408	-0.35678	Non-Mutant	NA	0.537341	0.547864	-0.01052	Stage IB	53.32544
7539	2.690967	2.043966	-0.647	Non-Mutant	NA	0.244566	0.430098	-0.18553	Stage IIA	26.00519
7540	2.685925	2.254683	-0.43124	Non-Mutant	NA	0.46671	0.643209	-0.1765	Stage IB	39.35299
7542	2.507458	2.176008	-0.33145	Non-Mutant	NA	0.672271	0.506081	0.16619	Stage IB	10.55331

7546	2.782297	2.120973	-0.66132	Non-Mutant	NA	0.304286	0.855165	-0.55088	Stage IA	42.24611
7547	2.709455	2.148728	-0.56073	Non-Mutant	NA	0.288964	0.68007	-0.39111	Stage IB	64.60203
7552	2.81392	2.273776	-0.54014	Non-Mutant	NA	0.47471	0.857295	-0.38259	Stage IB	63.51711
7553	2.883059	2.236873	-0.64619	Non-Mutant	NA	0.235555	0.98872	-0.75317	Stage IA	61.47878
7554	2.770642	2.197576	-0.57307	Non-Mutant	NA	0.533111	0.706744	-0.17363	Stage IIIA	25.47917
7562	2.577341	2.223182	-0.35416	Non-Mutant	NA	0.63994	0.458138	0.181802	Stage IIA	2.860243
7567	2.638172	2.189798	-0.44837	Non-Mutant	NA	0.605811	0.588938	0.016873	Stage IIB	18.67377
7570	2.154596	2.319362	0.164766	Non-Mutant	NA	0.585466	0.49649	0.088975	Stage IA	27.09011
7573	2.866965	2.210735	-0.65623	Non-Mutant	NA	0.488742	0.786272	-0.29753	Stage IA	16.01078
7574	2.802834	2.359683	-0.44315	Non-Mutant	NA	0.357735	0.834871	-0.47714	Stage IB	32.71197
7576	2.739353	2.176874	-0.56248	Non-Mutant	NA	0.475818	0.525267	-0.04945	Stage IB	22.02716
7624	2.287713	2.190126	-0.09759	Non-Mutant	NA	0.446776	0.642952	-0.19618	Stage IV	34.29004
7626	2.851492	2.140347	-0.71114	Non-Mutant	NA	0.330389	0.818676	-0.48829	Stage IIA	30.54213
7633	2.497984	2.408393	-0.08959	Mutant	G333C	0.423029	0.768461	-0.34543	Stage IB	50.23507
7659	2.752965	2.368068	-0.3849	Non-Mutant	NA	0.42015	0.628611	-0.20846	Stage IA	22.71756
7660	2.418889	2.360631	-0.05826	Non-Mutant	NA	0.426427	0.36275	0.063677	Stage IB	19.4628
7661	2.776291	2.116803	-0.65949	Non-Mutant	NA	0.387239	0.78133	-0.39409	Stage IB	18.31213
7662	2.53929	2.139201	-0.40009	Non-Mutant	NA	0.50799	0.558949	-0.05096	Stage IB	7.167045
7667	2.113464	2.4428	0.329337	Non-Mutant	NA	0.60262	0.385576	0.217044	Stage IIB	36.06536
7669	2.620513	2.463299	-0.15721	Non-Mutant	NA	0.48778	0.596436	-0.10866	Stage IIA	18.87103
7670	2.64052	2.420219	-0.2203	Non-Mutant	NA	0.654952	0.656099	-0.00115	Stage IIA	28.99694
7671	2.608354	2.628234	0.019879	Mutant	P278S	0.52054	0.688883	-0.16834	Stage IB	29.22708
7672	2.783134	2.395643	-0.38749	Non-Mutant	NA	0.604591	0.676099	-0.07151	Stage IA	23.6381
7701	2.562822	2.583884	0.021062	Non-Mutant	NA	0.442794	0.483965	-0.04117	Stage IV	31.13391
7711	2.459618	2.221734	-0.23788	Non-Mutant	NA	0.546677	0.635058	-0.08838	Stage IIA	34.38866
7713	2.400061	2.599747	0.199686	Non-Mutant	NA	0.506312	0.606602	-0.10029	Stage IIA	38.03794
7714	2.787071	2.18909	-0.59798	Non-Mutant	NA	0.241366	0.610772	-0.36941	Stage IIIA	20.54772

7724	2.758936	2.36131	-0.39763	Non-Mutant	NA	0.360672	0.747805	-0.38713	Stage IB	23.17783
7725	2.749548	2.104359	-0.64519	Non-Mutant	NA	0.281943	0.639091	-0.35715	Stage IA	14.53135
7726	2.495159	2.237428	-0.25773	Non-Mutant	NA	0.630548	0.633964	-0.00342	Stage IA	21.43538
7727	2.686141	2.292294	-0.39385	Non-Mutant	NA	0.388169	0.581752	-0.19358	Stage IIIA	3.912286
7728	2.782238	2.302194	-0.48004	Non-Mutant	NA	0.443625	0.774106	-0.33048	Stage IB	23.14495
7760	2.466978	2.133729	-0.33325	Non-Mutant	NA	0.501615	0.305968	0.195647	Stage IIB	6.641023
7761	2.609879	2.119461	-0.49042	Non-Mutant	NA	0.540134	0.71877	-0.17864	Stage IB	6.115001
7763	2.750835	2.506804	-0.24403	Non-Mutant	NA	0.568226	0.808437	-0.24021	Stage IA	22.68468
7764	2.596553	2.466802	-0.12975	Non-Mutant	NA	0.596952	0.642736	-0.04578	Stage IA	13.61081
7765	2.800763	2.240947	-0.55982	Non-Mutant	NA	0.40256	0.616495	-0.21393	Stage IV	5.424598
7771	2.744298	2.470654	-0.27364	Non-Mutant	NA	0.674399	0.848768	-0.17437	Stage IIB	16.17517
7813	2.462183	2.491393	0.02921	Non-Mutant	NA	0.675126	0.428055	0.247072	Stage IIIB	13.93957
7815	2.378054	2.536553	0.158499	Mutant	A191T	0.563174	0.491517	0.071657	Stage IB	25.41342
7816	2.672017	2.191497	-0.48052	Non-Mutant	NA	0.221412	1.013512	-0.7921	Stage IV	15.38613
7903	2.708492	2.092484	-0.61601	Non-Mutant	NA	0.489413	0.591924	-0.10251	Stage IA	18.64089
7907	2.642493	2.254708	-0.38778	Non-Mutant	NA	0.386074	0.491175	-0.1051	Stage IIA	11.27659
7910	2.409437	2.528145	0.118708	Non-Mutant	NA	0.415733	0.152735	0.262997	Stage IIA	34.19141
7911	2.764353	2.150006	-0.61435	Non-Mutant	NA	0.422014	0.643551	-0.22154	Stage IA	17.6546
7913	2.349364	2.521952	0.172588	Non-Mutant	NA	0.615767	0.324591	0.291176	Stage IA	18.44363
7914	2.699522	2.047602	-0.65192	Non-Mutant	NA	0.590477	0.833342	-0.24287	Stage IIA	6.147878
7937	2.617322	2.071948	-0.54537	Non-Mutant	NA	0.332445	0.345504	-0.01306	Stage IB	18.54226
7938	2.771061	2.349619	-0.42144	Non-Mutant	NA	0.261557	0.692633	-0.43108	Stage IA	0.591774
7941	2.820357	2.352575	-0.46778	Non-Mutant	NA	0.421464	0.795458	-0.37399	Stage IA	15.91215
7944	2.505021	2.267715	-0.23731	Non-Mutant	NA	0.575891	0.773949	-0.19806	Stage IA	12.39438
7947	2.716657	2.12255	-0.59411	Mutant	S102L	0.316103	0.718387	-0.40228	Stage IA	15.68202
7948	2.569182	2.038329	-0.53085	Non-Mutant	NA	0.491947	0.687199	-0.19525	Stage IB	15.64914
7953	2.661066	2.0835	-0.57757	Non-Mutant	NA	0.400805	0.635607	-0.2348	Stage IA	32.77772

7954	2.804726	2.324506	-0.48022	Non-Mutant	NA	0.473251	0.732774	-0.25952	Stage IB	19.89019
7955	2.030646	2.459072	0.428426	Non-Mutant	NA	0.424541	0.380814	0.043727	Stage IB	35.24345
7973	2.580273	2.610866	0.030594	Non-Mutant	NA	0.615117	0.315269	0.299848	Stage IB	7.561561
7974	2.819155	2.262646	-0.55651	Non-Mutant	NA	0.373396	0.678871	-0.30547	Stage IIIA	6.049249
7978	2.745766	2.145988	-0.59978	Non-Mutant	NA	0.422485	0.645543	-0.22306	Stage IIB	4.405431
7979	2.529534	2.286349	-0.24318	Non-Mutant	NA	0.624706	0.520636	0.104069	Stage IB	13.41355
7980	2.727146	2.269892	-0.45725	Non-Mutant	NA	0.578655	0.789656	-0.211	Stage I	13.51218
7994	2.679742	2.162274	-0.51747	Non-Mutant	NA	0.525337	0.888315	-0.36298	Stage IIB	19.82444
7995	2.695478	2.23016	-0.46532	Non-Mutant	NA	0.309211	0.779437	-0.47023	Stage IA	29.22708
8025	2.678841	2.392409	-0.28643	Non-Mutant	NA	0.445604	0.550381	-0.10478	Stage IIIA	34.84893
8028	2.85592	2.241783	-0.61414	Non-Mutant	NA	0.341104	0.605522	-0.26442	Stage IA	36.75576
8032	2.563371	2.681505	0.118134	Non-Mutant	NA	0.381916	0.408974	-0.02706	Stage IA	1.446559
8033	2.647906	2.388997	-0.25891	Non-Mutant	NA	0.525436	0.664622	-0.13919	Stage IV	21.56689
8039	2.836729	2.202592	-0.63414	Non-Mutant	NA	0.35552	0.670897	-0.31538	Stage IA	27.28737
8054	2.319737	2.552486	0.232749	Non-Mutant	NA	0.496656	0.383116	0.11354	Stage IIB	37.74205
8055	2.726515	2.221878	-0.50464	Non-Mutant	NA	0.569647	0.638008	-0.06836	Stage IIA	4.076668
8056	2.755783	2.186679	-0.5691	Non-Mutant	NA	0.231125	0.833134	-0.60201	Stage IIIA	4.569813
8067	2.585061	2.515517	-0.06954	Non-Mutant	NA	0.498838	0.504907	-0.00607	Stage IB	6.115001
8073	2.722916	2.350537	-0.37238	Non-Mutant	NA	0.286294	0.693316	-0.40702	Stage IB	24.3285
8074	2.766146	2.139001	-0.62714	Non-Mutant	NA	0.550568	0.575659	-0.02509	Stage IIA	0.789032
8075	2.733497	2.175952	-0.55755	Non-Mutant	NA	0.634126	0.57845	0.055676	Stage IB	22.81619
8076	2.820431	2.441265	-0.37917	Non-Mutant	NA	0.36762	0.792065	-0.42444	Stage IA	32.64622
8085	2.67514	2.40206	-0.27308	Non-Mutant	NA	0.617575	0.760326	-0.14275	Stage IA	29.72022
8087	2.70665	2.140016	-0.56663	Non-Mutant	NA	0.409228	0.671949	-0.26272	Stage IB	15.18887
8089	2.714639	2.289114	-0.42552	Non-Mutant	NA	0.365704	0.727982	-0.36228	Stage IA	23.0792
8090	2.768529	2.250722	-0.51781	Non-Mutant	NA	0.510259	0.529208	-0.01895	Stage IA	19.66006
8091	2.773487	2.305333	-0.46815	Non-Mutant	NA	0.443723	0.550385	-0.10666	Stage IB	19.72581

8092	2.574969	2.339226	-0.23574	Non-Mutant	NA	0.61127	0.66273	-0.05146	Stage IIB	5.062958
8094	2.216562	2.648678	0.432115	Non-Mutant	NA	0.593655	0.335273	0.258382	Stage IV	17.78611
8096	2.744164	2.206994	-0.53717	Non-Mutant	NA	0.496426	0.586109	-0.08968	Stage IB	23.6381
8097	2.780716	2.187127	-0.59359	Non-Mutant	NA	0.328298	0.774864	-0.44657	Stage IA	15.64914
8117	2.617098	2.061494	-0.5556	Non-Mutant	NA	0.204361	0.607493	-0.40313	Stage IB	12.6574
8119	2.531068	2.528752	-0.00232	Non-Mutant	NA	0.519727	0.491372	0.028355	Stage IIB	9.36976
8120	2.661681	2.234694	-0.42699	Non-Mutant	NA	0.414222	0.693464	-0.27924	Stage IB	8.547852
8171	2.557754	2.02124	-0.53651	Non-Mutant	NA	0.513523	0.551155	-0.03763	Stage IV	18.67377
8172	2.794978	2.242424	-0.55255	Non-Mutant	NA	0.344783	0.826935	-0.48215	Stage IB	17.91761
8174	2.728387	2.424714	-0.30367	Non-Mutant	NA	0.306788	0.954663	-0.64788	Stage IIA	5.391722
8175	2.735429	2.313899	-0.42153	Non-Mutant	NA	0.23986	0.453437	-0.21358	Stage IB	18.11487
8176	2.682514	2.565672	-0.11684	Non-Mutant	NA	0.539434	0.516461	0.022973	Stage IIIA	15.38613
8177	2.857465	2.328761	-0.5287	Non-Mutant	NA	0.405612	0.491906	-0.08629	Stage IB	16.4053
8179	2.630036	2.558061	-0.07197	Non-Mutant	NA	0.562007	0.835486	-0.27348	Stage IA	14.30121
8192	2.837218	2.286348	-0.55087	Non-Mutant	NA	0.409365	0.532824	-0.12346	Stage IIA	24.29562
8194	2.640037	2.487351	-0.15269	Non-Mutant	NA	0.429937	0.74214	-0.3122	Stage IIB	23.80248
8203	2.671574	2.613438	-0.05814	Non-Mutant	NA	0.719223	0.544909	0.174313	Stage IA	17.98336
8204	2.602572	2.351534	-0.25104	Non-Mutant	NA	0.556508	0.715773	-0.15926	Stage IB	16.93132
8205	2.761107	2.059164	-0.70194	Non-Mutant	NA	0.327861	0.877137	-0.54928	Stage IIA	19.69293
8206	2.799959	2.253386	-0.54657	Non-Mutant	NA	0.272637	0.768458	-0.49582	Stage IA	29.1942
8207	2.758913	2.257697	-0.50122	Non-Mutant	NA	0.39414	0.676036	-0.2819	Stage IB	32.1202
8208	2.829904	2.386788	-0.44312	Non-Mutant	NA	0.584433	0.823788	-0.23936	Stage IA	22.15866
8253	2.73622	2.444168	-0.29205	Non-Mutant	NA	0.33264	0.541899	-0.20926	Stage IIA	14.00533
8255	2.46246	2.442692	-0.01977	Non-Mutant	NA	0.374577	0.650324	-0.27575	Stage IA	4.241049
8278	2.751293	2.146338	-0.60496	Non-Mutant	NA	0.509052	0.524551	-0.0155	Stage IIB	31.03528
8279	2.641442	2.124355	-0.51709	Non-Mutant	NA	0.454195	0.485768	-0.03157	Stage IIA	31.19966
8280	2.865105	2.188969	-0.67614	Non-Mutant	NA	0.470796	0.755122	-0.28433	Stage IIA	23.04632

8281	2.604734	2.50587	-0.09886	Non-Mutant	NA	0.3584	0.708343	-0.34994	Stage IA	0
8299	2.786762	2.435906	-0.35086	Non-Mutant	NA	0.347427	0.901308	-0.55388	Stage IA	15.41901
8301	2.764601	2.133435	-0.63117	Non-Mutant	NA	0.425346	0.743121	-0.31777	Stage IB	17.55597
8302	2.719816	2.209772	-0.51004	Non-Mutant	NA	0.518225	0.481524	0.036701	Stage IB	15.7149
8358	2.259756	2.336074	0.076318	Non-Mutant	NA	0.592979	0.449946	0.143033	Stage IB	21.46826
8359	2.562973	2.519906	-0.04307	Non-Mutant	NA	0.545121	0.473327	0.071794	Stage IIIA	14.5971
8394	2.756562	2.249081	-0.50748	Non-Mutant	NA	0.548706	0.436785	0.111922	Stage IIIB	4.569813
8395	2.67971	2.35911	-0.3206	Non-Mutant	NA	0.634386	0.778392	-0.14401	Stage IIB	39.97764
8397	2.739025	2.08992	-0.6491	Non-Mutant	NA	0.429752	0.525669	-0.09592	Stage IIB	42.37762
8398	2.567272	2.564688	-0.00258	Non-Mutant	NA	0.404088	0.525941	-0.12185	Stage IIIA	14.5971
8399	2.600666	2.348995	-0.25167	Non-Mutant	NA	0.536364	0.730084	-0.19372	Stage IIIA	88.63465
8402	2.559063	2.360136	-0.19893	Non-Mutant	NA	0.600085	0.558768	0.041318	Stage IIIA	49.24878
8453	2.845304	2.374573	-0.47073	Non-Mutant	NA	0.486407	0.814352	-0.32795	Stage IIB	26.72847
8457	2.819519	2.162796	-0.65672	Non-Mutant	NA	0.305858	0.853552	-0.54769	Stage IA	36.9859
8459	2.705943	2.291852	-0.41409	Non-Mutant	NA	0.422007	0.832303	-0.4103	Stage IIB	36.78864
8460	2.796011	2.129164	-0.66685	Non-Mutant	NA	0.473669	0.643684	-0.17001	Stage IA	27.2545
8494	2.627125	2.021089	-0.60604	Non-Mutant	NA	0.291484	0.328937	-0.03745	Stage IIA	2.761614
8496	2.807522	2.126554	-0.68097	Non-Mutant	NA	0.185811	0.526299	-0.34049	Stage IB	16.60256
8497	2.839033	2.509526	-0.32951	Non-Mutant	NA	0.55212	0.865849	-0.31373	Stage IA	14.26834
8499	2.306654	1.982794	-0.32386	Non-Mutant	NA	0.62203	0.398198	0.223832	Stage IA	1.183549
8505	2.449271	2.59427	0.144999	Non-Mutant	NA	0.597216	0.58051	0.016706	Stage IIIA	14.46559
8506	2.703775	2.20818	-0.49559	Non-Mutant	NA	0.390349	0.412409	-0.02206	Stage IIB	0.36164
8507	2.763473	2.453341	-0.31013	Non-Mutant	NA	0.494379	0.612188	-0.11781	Stage IA	13.74232
8508	2.583062	2.614464	0.031402	Non-Mutant	NA	0.66265	0.493822	0.168827	Stage IIA	20.28471
8510	2.865469	2.250137	-0.61533	Non-Mutant	NA	0.261643	0.781019	-0.51938	Stage IB	17.72035
8511	2.722103	2.281315	-0.44079	Non-Mutant	NA	0.397082	0.522664	-0.12558	Stage IB	18.14775
8512	2.72853	2.530685	-0.19785	Non-Mutant	NA	0.367409	0.825421	-0.45801	Stage IV	19.95595

8513	2.869062	2.300164	-0.5689	Non-Mutant	NA	0.246501	0.885925	-0.63942	Stage IIB	26.00519
8514	2.744076	2.073578	-0.6705	Non-Mutant	NA	0.405187	0.494275	-0.08909	Stage IB	17.0957
8520	2.702436	2.005341	-0.69709	Non-Mutant	NA	0.567259	0.469027	0.098232	Stage IB	6.904034
8547	2.742636	2.258025	-0.48461	Non-Mutant	NA	0.549765	0.532685	0.01708	Stage IIIA	21.59976
8552	2.86464	2.242423	-0.62222	Non-Mutant	NA	0.275276	0.702219	-0.42694	Stage I	20.5806
8585	2.676257	2.421848	-0.25441	Non-Mutant	NA	0.286884	0.362017	-0.07513	Stage IB	11.60535
8614	2.698814	2.214132	-0.48468	Non-Mutant	NA	0.570712	0.58514	-0.01443	Stage IB	17.62172
8615	2.370393	2.641597	0.271204	Non-Mutant	NA	0.521229	0.36219	0.159039	Stage IIIA	14.66285
8616	2.659439	2.198857	-0.46058	Non-Mutant	NA	0.345578	0.492056	-0.14648	Stage IB	1.578065
8619	2.859445	2.269161	-0.59028	Non-Mutant	NA	0.331986	0.95507	-0.62308	Stage IIB	13.67656
8620	2.383885	2.315759	-0.06813	Non-Mutant	NA	0.380363	0.497061	-0.1167	Stage IV	12.32863
8621	2.885352	2.325126	-0.56023	Non-Mutant	NA	0.299588	0.973168	-0.67358	Stage IA	16.93132
8640	2.638788	2.331871	-0.30692	Non-Mutant	NA	0.39675	0.578443	-0.18169	Stage IIA	232.1728
8648	2.820633	2.128584	-0.69205	Non-Mutant	NA	0.392191	0.881607	-0.48942	Stage IIB	39.74751
8655	2.781269	2.245891	-0.53538	Non-Mutant	NA	0.401733	0.658083	-0.25635	Stage IA	77.58819
8660	2.750519	2.395717	-0.3548	Non-Mutant	NA	0.32495	0.672921	-0.34797	Stage IIB	10.55331
8662	2.381607	2.193504	-0.1881	Non-Mutant	NA	0.285425	0.486518	-0.20109	Stage IB	110.4974
8668	2.839354	2.112769	-0.72659	Non-Mutant	NA	0.358004	0.819659	-0.46166	Stage IA	13.9067
8669	2.623186	2.334067	-0.28912	Non-Mutant	NA	0.455456	0.635591	-0.18013	Stage IA	30.83802
8671	2.869152	2.126113	-0.74304	Non-Mutant	NA	0.405171	0.984415	-0.57924	Stage IIB	27.58326
8672	2.610947	2.466508	-0.14444	Non-Mutant	NA	0.646255	0.608477	0.037777	Stage IIB	0.624651
8673	2.549789	2.303064	-0.24673	Non-Mutant	NA	0.427136	0.462638	-0.0355	Stage IB	28.33942
8674	2.402473	2.537291	0.134818	Non-Mutant	NA	0.548426	0.459654	0.088772	Stage IIA	26.49834
A443	2.723568	2.429158	-0.29441	Non-Mutant	NA	0.500775	0.462465	0.03831	Stage IA	6.345136
A444	2.759692	2.104832	-0.65486	Non-Mutant	NA	0.350809	0.771636	-0.42083	Stage IA	10.09304
A44F	2.823138	2.368097	-0.45504	Non-Mutant	NA	0.494326	0.684109	-0.18978	Stage IB	4.372555
A456	2.843297	2.251708	-0.59159	Non-Mutant	NA	0.324568	0.732254	-0.40769	Stage IA	29.45721

A46O	1.885352	2.55285	0.667498	Non-Mutant	NA	0.662167	0.334532	0.327635	Stage IB	47.80222
A46P	2.573052	2.428615	-0.14444	Non-Mutant	NA	0.473634	0.426653	0.046981	Stage IB	19.52855
A46R	2.670991	2.095303	-0.57569	Non-Mutant	NA	0.349488	0.604089	-0.2546	Stage IB	56.71171
A46S	2.648529	2.50924	-0.13929	Non-Mutant	NA	0.609381	0.661125	-0.05174	Stage IB	54.34461
A46U	2.750132	2.183775	-0.56636	Non-Mutant	NA	0.494573	0.780457	-0.28588	Stage IIB	67.95542
A46V	2.710957	2.017954	-0.693	Non-Mutant	NA	0.569237	0.653463	-0.08423	Stage IB	72.2951
A46Y	2.859188	2.171504	-0.68768	Non-Mutant	NA	0.314557	0.625321	-0.31076	Stage IIIA	13.61081
A470	2.712013	2.473943	-0.23807	Non-Mutant	NA	0.409853	0.480286	-0.07043	Stage IB	39.25436
A471	2.269232	2.567925	0.298693	Non-Mutant	NA	0.590435	0.388655	0.20178	Stage IIB	40.96393
A472	2.717971	2.196915	-0.52106	Non-Mutant	NA	0.290859	0.385957	-0.0951	Stage IIB	29.91748
A479	2.590626	2.490419	-0.10021	Non-Mutant	NA	0.605715	0.587508	0.018208	Stage IB	15.97791
A47A	2.752908	2.171156	-0.58175	Non-Mutant	NA	0.451491	0.391534	0.059957	Stage IB	15.32038
A47B	2.666801	2.179056	-0.48775	Non-Mutant	NA	0.373954	0.512008	-0.13805	Stage IB	9.435513
A47G	2.872697	2.270769	-0.60193	Non-Mutant	NA	0.444503	0.644912	-0.20041	Stage IA	11.5396
A48X	2.831718	2.29071	-0.54101	Non-Mutant	NA	0.422274	0.737417	-0.31514	Stage IIA	22.65181
A48Y	2.536703	2.460399	-0.0763	Non-Mutant	NA	0.524957	0.65421	-0.12925	Stage IIA	20.7121
A48Z	2.709809	2.267844	-0.44197	Non-Mutant	NA	0.399019	0.478833	-0.07981	Stage IIIB	21.40251
A490	2.526706	2.049092	-0.47761	Non-Mutant	NA	0.51035	0.575261	-0.06491	Stage IIA	3.254759
A491	2.665847	2.22961	-0.43624	Non-Mutant	NA	0.457027	0.668565	-0.21154	Stage IA	20.5806
A492	2.587225	2.642208	0.054982	Non-Mutant	NA	0.451843	0.559476	-0.10763	Stage IA	19.59431
A493	2.600267	2.014572	-0.5857	Non-Mutant	NA	0.410664	0.411778	-0.00111	Stage IB	0.920538
A494	2.115867	2.375333	0.259466	Non-Mutant	NA	0.694046	0.359539	0.334508	Stage IB	15.81353
A4AD	2.56262	2.373852	-0.18877	Non-Mutant	NA	0.472399	0.485882	-0.01348	Stage IA	18.08199
A4AE	2.837475	2.125444	-0.71203	Non-Mutant	NA	0.321558	0.793562	-0.472	Stage IA	35.47358
A4AG	2.79905	2.152757	-0.64629	Non-Mutant	NA	0.49701	0.605388	-0.10838	Stage IA	32.48184
A4BC	2.492678	2.480056	-0.01262	Non-Mutant	NA	0.582472	0.704506	-0.12203	Stage IIA	1.446559
A4BD	2.803478	2.267113	-0.53637	Non-Mutant	NA	0.345086	0.524854	-0.17977	Stage IIA	19.82444

A4D0	2.203835	2.566271	0.362435	Non-Mutant	NA	0.557216	0.164379	0.392837	Stage IIA	3.813657
A4DF	2.630359	2.282532	-0.34783	Non-Mutant	NA	0.371013	0.559179	-0.18817	Stage IA	20.18608
A4DG	2.768494	2.267933	-0.50056	Non-Mutant	NA	0.286263	0.803594	-0.51733	Stage IA	19.98882
A4E5	2.596068	2.120122	-0.47595	Non-Mutant	NA	0.412144	0.570514	-0.15837	Stage I	19.00253
A4E6	2.816563	2.100098	-0.71647	Non-Mutant	NA	0.048867	1.006639	-0.95777	Stage IA	14.30121
A4EZ	2.30934	2.259206	-0.05013	Non-Mutant	NA	0.602035	0.451471	0.150564	Stage IIA	35.21057
A4JF	2.765967	2.23061	-0.53536	Non-Mutant	NA	0.470191	0.441235	0.028956	Stage IIB	24.22987
A4JN	2.732783	2.122551	-0.61023	Non-Mutant	NA	0.579018	0.69868	-0.11966	Stage IV	23.60522
A4JO	2.797429	2.192808	-0.60462	Non-Mutant	NA	0.230923	0.70441	-0.47349	Stage IA	1.08492
A4JP	2.807124	2.276274	-0.53085	Non-Mutant	NA	0.45726	1.017642	-0.56038	Stage IV	19.00253
A4JQ	2.814435	2.237607	-0.57683	Non-Mutant	NA	0.363707	0.60898	-0.24527	Stage IA	17.29296
A4LX	2.819938	2.101469	-0.71847	Non-Mutant	NA	0.365807	0.789532	-0.42372	Stage IB	20.18608
A4M0	2.820587	2.140421	-0.68017	Non-Mutant	NA	0.248015	0.696209	-0.44819	Stage IB	21.43538
A4M1	2.842398	2.184879	-0.65752	Non-Mutant	NA	0.168328	0.827801	-0.65947	Stage IA	19.75869
A4M2	2.843696	2.411749	-0.43195	Non-Mutant	NA	0.164592	0.924506	-0.75991	Stage IA	20.51484
A4M3	2.615201	2.495013	-0.12019	Non-Mutant	NA	0.354955	0.57648	-0.22153	Stage IA	17.75323
A4M5	2.790579	2.492116	-0.29846	Non-Mutant	NA	0.378275	0.792192	-0.41392	Stage IA	20.84361
A4M6	2.830425	2.149504	-0.68092	Non-Mutant	NA	0.242492	0.672667	-0.43017	Stage IA	18.67377
A4M7	2.841908	2.245624	-0.59628	Non-Mutant	NA	0.459881	0.678186	-0.2183	Stage IA	20.67923
A4N1	2.509724	2.289465	-0.22026	Non-Mutant	NA	0.4528	0.275982	0.176818	Stage IIA	27.18874
A4N4	2.527344	2.32356	-0.20378	Non-Mutant	NA	0.432731	0.487824	-0.05509	Stage IA	38.62971
A4N5	2.761212	2.107072	-0.65414	Non-Mutant	NA	0.389021	0.744112	-0.35509	Stage IA	2.761614
A4P7	2.844461	2.134292	-0.71017	Non-Mutant	NA	0.245239	0.816363	-0.57112	Stage IB	13.64369
A4P8	2.857481	2.014295	-0.84319	Non-Mutant	NA	-0.09879	1.015209	-1.114	Stage IIIA	26.46546
A4SS	2.597198	2.234082	-0.36312	Non-Mutant	NA	0.378288	0.710548	-0.33226	Stage IA	13.64369
A4SU	2.720452	2.305169	-0.41528	Non-Mutant	NA	0.41802	0.608369	-0.19035	Stage IA	13.44643
A4SV	2.756735	2.318144	-0.43859	Non-Mutant	NA	0.461216	0.571832	-0.11062	Stage IB	86.13604

A4SW	2.832915	2.245641	-0.58727	Non-Mutant	NA	0.473106	0.497922	-0.02482	Stage IIB	58.45415
A4SY	2.71438	2.358378	-0.356	Non-Mutant	NA	0.42972	0.351276	0.078444	Stage IIB	49.3474
A4T4	2.798778	2.15372	-0.64506	Non-Mutant	NA	0.568618	0.679632	-0.11101	Stage IIB	86.03741
A4T6	2.730103	2.141598	-0.58851	Non-Mutant	NA	0.468232	0.596429	-0.1282	Stage IIIA	58.84867
A4T7	2.756611	2.643668	-0.11294	Non-Mutant	NA	0.401753	0.631201	-0.22945	Stage IV	5.490351
A4T8	2.392004	2.581423	0.189419	Non-Mutant	NA	0.702629	0.410986	0.291643	Stage IIIA	5.293093
A4T9	2.819107	2.233034	-0.58607	Non-Mutant	NA	0.362536	0.632761	-0.27023	Stage IIIA	41.58859
A4TA	2.551547	2.586185	0.034637	Non-Mutant	NA	0.589388	0.532167	0.057221	Stage IA	31.23253
A4TC	2.694494	2.267827	-0.42667	Non-Mutant	NA	0.414454	0.467097	-0.05264	Stage IIIA	2.43285
A4TD	2.595076	2.653022	0.057947	Non-Mutant	NA	0.522665	0.604042	-0.08138	Stage IIIA	10.09304
A4TE	2.128989	2.712421	0.583433	Non-Mutant	NA	0.324121	0.327029	-0.00291	Stage IIA	29.45721
A4TF	2.451248	2.329073	-0.12217	Non-Mutant	NA	0.408002	0.394066	0.013937	Stage IIA	11.04645
A4TH	2.786444	2.259383	-0.52706	Non-Mutant	NA	0.497158	0.84365	-0.34649	Stage IA	24.36138
A4TI	2.765603	2.103064	-0.66254	Non-Mutant	NA	0.311198	0.727472	-0.41627	Stage IIA	14.10396
A4TJ	2.827517	2.487209	-0.34031	Non-Mutant	NA	0.569091	0.723863	-0.15477	Stage IA	11.14508
A4TK	2.765524	2.171611	-0.59391	Non-Mutant	NA	0.429457	0.580087	-0.15063	Stage IIB	19.13404
A4VK	2.61537	2.506383	-0.10899	Non-Mutant	NA	0.519202	0.608029	-0.08883	Stage IIIA	21.40251
A4VN	2.810782	2.232039	-0.57874	Non-Mutant	NA	0.429491	0.624773	-0.19528	Stage IIA	18.18062
A4VP	2.706229	2.632707	-0.07352	Non-Mutant	NA	0.476319	0.61995	-0.14363	Stage IIIA	19.89019
A4YF	2.280666	2.43374	0.153075	Non-Mutant	NA	0.532079	0.435023	0.097057	Stage IA	71.0458
A4YG	2.769489	2.430183	-0.33931	Non-Mutant	NA	0.552909	0.542449	0.01046	Stage IB	74.33343
A4YI	2.788675	2.280059	-0.50862	Non-Mutant	NA	0.328622	0.457101	-0.12848	Stage IIIA	0.131505
A4YP	2.639907	2.573176	-0.06673	Non-Mutant	NA	0.673504	0.575493	0.098011	Stage IB	1.643818
A4YQ	2.658245	2.288944	-0.3693	Non-Mutant	NA	0.183603	0.648469	-0.46487	Stage IA	47.07894
A50W	2.749411	2.123646	-0.62577	Non-Mutant	NA	0.468299	0.519864	-0.05157	Stage IIA	14.53135
A52J	2.797434	1.955509	-0.84192	Non-Mutant	NA	0.374825	0.83248	-0.45766	Stage IA	59.11168
A55A	2.812884	2.254605	-0.55828	Non-Mutant	NA	0.428068	0.72547	-0.2974	Stage IB	0.493145

A55O	2.788085	2.554636	-0.23345	Non-Mutant	NA	0.569035	0.700665	-0.13163	Stage IIA	0.427393
A55R	2.541139	2.570964	0.029826	Non-Mutant	NA	0.43957	0.57541	-0.13584	Stage IA	19.82444
A57B	2.798533	2.034109	-0.76442	Non-Mutant	NA	0.314486	0.770537	-0.45605	Stage IA	17.95049
A59K	2.691109	2.158907	-0.5322	Non-Mutant	NA	0.257701	0.489903	-0.2322	Stage IIB	19.42992
A5C7	2.46778	2.38293	-0.08485	Non-Mutant	NA	0.557923	0.618469	-0.06055	Stage IB	73.90604
A5IP	2.363895	2.495082	0.131187	Non-Mutant	NA	0.480242	0.616965	-0.13672	Stage IV	1.906828
A743	2.853217	2.275273	-0.57794	Non-Mutant	NA	0.37642	0.649442	-0.27302	Stage IIA	21.8299
A7SV	2.43036	1.958836	-0.47152	Non-Mutant	NA	0.465906	0.754073	-0.28817	Stage IIA	18.57514
A7XG	2.561126	2.348939	-0.21219	Non-Mutant	NA	0.231189	0.493148	-0.26196	Stage IIIA	20.28471
A8F4	2.567621	2.352279	-0.21534	Non-Mutant	NA	0.506633	0.585444	-0.07881	Stage IB	15.64914
A93V	2.333223	2.482213	0.14899	Non-Mutant	NA	0.475223	0.375957	0.099265	Stage IA	9.862906
A9RS	2.310073	2.463832	0.153759	Non-Mutant	NA	0.469005	0.399643	0.069362	Stage IIB	11.17796
AA1A	2.71695	2.178204	-0.53875	Non-Mutant	NA	0.620469	0.769185	-0.14872	Stage IA	16.86557
AA5R	2.834195	2.162096	-0.6721	Non-Mutant	NA	0.114376	0.951212	-0.83684	Stage IA	21.63264
AAQV	2.73321	2.355653	-0.37756	Non-Mutant	NA	0.49394	0.524489	-0.03055	Stage II	22.25729
AAR0	2.798654	2.62666	-0.17199	Non-Mutant	NA	0.281008	0.584263	-0.30325	Stage IA	156.6558
AAR2	2.527366	2.398615	-0.12875	Non-Mutant	NA	0.658607	0.504988	0.15362	Stage IB	73.11701
AAR3	2.584056	2.212456	-0.3716	Non-Mutant	NA	0.587457	0.598136	-0.01068	Stage IIB	62.23493
AAR4	2.669087	2.212078	-0.45701	Non-Mutant	NA	0.409992	0.605741	-0.19575	Stage IIIA	28.89831
AAR9	2.149117	2.259839	0.110722	Non-Mutant	NA	0.491955	0.381974	0.109981	Stage IIB	8.547852
AARE	2.549364	2.388627	-0.16074	Non-Mutant	NA	0.393152	0.377556	0.015595	Stage IA	40.40504
AARN	2.807213	2.32317	-0.48404	Non-Mutant	NA	0.61218	0.467717	0.144463	Stage IA	37.31466
AARO	2.78835	2.27432	-0.51403	Non-Mutant	NA	0.481126	0.544475	-0.06335	Stage IA	123.5822
AARQ	2.534773	2.100594	-0.43418	Non-Mutant	NA	0.557798	0.429498	0.128299	Stage I	221.3236
AARR	2.795047	2.380615	-0.41443	Non-Mutant	NA	0.406125	0.904995	-0.49887	Stage IA	164.1187
